# Intradialytic hypotension prediction using covariance matrix-driven whale optimizer with orthogonal structure-assisted extreme learning machine

**DOI:** 10.3389/fninf.2022.956423

**Published:** 2022-10-31

**Authors:** Yupeng Li, Dong Zhao, Guangjie Liu, Yi Liu, Yasmeen Bano, Alisherjon Ibrohimov, Huiling Chen, Chengwen Wu, Xumin Chen

**Affiliations:** ^1^College of Computer Science and Technology, Changchun Normal University, Changchun, China; ^2^Department of Nephrology, The First Affiliated Hospital of Wenzhou Medical University, Wenzhou, China; ^3^College of Computer Science and Artificial Intelligence, Wenzhou University, Wenzhou, China; ^4^Department of Nephrology, The First Affiliated Hospital of Wenzhou Medical University, Wenzhou University, Wenzhou, China

**Keywords:** medical diagnosis, machine learning, swarm intelligence, feature selection, intradialytic hypotension

## Abstract

Intradialytic hypotension (IDH) is an adverse event occurred during hemodialysis (HD) sessions with high morbidity and mortality. The key to preventing IDH is predicting its pre-dialysis and administering a proper ultrafiltration prescription. For this purpose, this paper builds a prediction model (bCOWOA-KELM) to predict IDH using indices of blood routine tests. In the study, the orthogonal learning mechanism is applied to the first half of the WOA to improve the search speed and accuracy. The covariance matrix is applied to the second half of the WOA to enhance the ability to get out of local optimum and convergence accuracy. Combining the above two improvement methods, this paper proposes a novel improvement variant (COWOA) for the first time. More, the core of bCOWOA-KELM is that the binary COWOA is utilized to improve the performance of the KELM. In order to verify the comprehensive performance of the study, the paper sets four types of comparison experiments for COWOA based on 30 benchmark functions and a series of prediction experiments for bCOWOA-KELM based on six public datasets and the HD dataset. Finally, the results of the experiments are analyzed separately in this paper. The results of the comparison experiments prove fully that the COWOA is superior to other famous methods. More importantly, the bCOWOA performs better than its peers in feature selection and its accuracy is 92.41%. In addition, bCOWOA improves the accuracy by 0.32% over the second-ranked bSCA and by 3.63% over the worst-ranked bGWO. Therefore, the proposed model can be used for IDH prediction with future applications.

## Introduction

End-stage renal disease (ESRD) threatens tens of millions of lives. Renal replacement therapy includes hemodialysis (HD), peritoneal dialysis (PD), and renal transplantation. Compared with transplantation, dialysis partially replaces renal function. Thus, there are several complications in dialysis patients despite intrinsic complications of ESRD, especially HD. HD is a treatment drawing blood out of patients, diffusing uremic toxins, ultrafiltering extra volume, and transfusion the purified blood back to the patient. The hemodynamics is unstable during HD. Once the patient’s cardiac function or peripheral vascular resistance cannot compensate, intradialytic hypotension (IDH) occurs.

IDH is defined according to different studies or guidelines. Even if systolic pressure (SBP) declines 20 mmHg without any symptoms, there are still target organ injuries and increased mortality (Burton et al., 2009a). Episodes of IDH decease perfusion to the heart, renal, brain, limbs, and mesenterium induces various complications. Examples are ischemic cardiomyopathy (Burton et al., 2009b), cerebral infarction (Naganuma et al., 2005), rapid loss of residual renal function (Jansen et al., 2002), critical limb ischemia (Matsuura et al., 2019), mesenteric ischemia (Ori et al., 2005), and vascular access thrombosis (Chang et al., 2011). The symptoms of IDH range from asymptomatic to loss of consciousness and sudden death. Therefore, managing IDH is an excellent way to avoid HD’s adverse events. When IDH episodes during HD, there are several acute managements, including administering saline, lowering the dialysate temperature and ultrafiltration rate, reducing the dialyzer blood flow, and increasing dialysate sodium concentration. Although physicians combine these treatments, dialysis treatment must be stopped in severe cases. In addition, the long-term benefits are still debated. Some studies reported that reduction of dialysate temperature prevented IDH, but meta-analysis showed the effect was uncertain. Furthermore, as compared to conventional dialysate, it may increase the rate of pain (Tsujimoto et al., 2019).

Schytz et al. (2015) performed a randomized clinical trial (RCT), and the results did not show any consistent trend in blood pressure (BP) changes to a reduction of the dialyzer blood flow. Sherman (2016) summarized the experience in their center; it was a common practice to lower the dialyzer blood flow in patients who developed IDH. However, the consideration did not apply to current dialysis practice. A meta-analysis reported that stepwise sodium profiling rather than linear sodium profiling effectively reduced IDH (Dunne, 2017). The results of sodium profiling were quick, and there was worry that in the long run, sodium profiling might result in a positive sodium balancing, increased thirst, and interdialytic weight increases (IDWG). ARCT showed low dialysate sodium concentration (135 mmol/L) significantly reduced IDWG, while no statistical difference in IDH episodes over 12 months of follow-up (Marshall et al., 2020). Radhakrishnan et al. (2020) showed there were lower IDWG, pre-HD SBP, and incidence of IDH when dialysate sodium concentration was equal to individual serum sodium level instead of high dialysate sodium concentration (140 mmol/L). Administration of saline and limited ultrafiltration rate prevent IDH by increasing relative blood volume, but always result in post-dialysis hypervolemia and heart failure. An inadequate ultrafiltration prescription induces IDH episodes, then nurses have to reducing ultrafiltration rate, leading to ultrafiltration failure in a 4-h dialysis session.

Artificial intelligence (AI), which focuses on modeling human cognition in computing, has achieved significant progress in a broad range of disciplines (Zhang J. et al., 2021; Luo et al., 2022). AI-assisted medical systems have recently gotten attention, making diagnosis systems and medical decision-making more instant, autonomous, and intelligent (Li et al., 2020c,^2022^; Zhang M. et al., 2021; Liu et al., 2022e). Thus, developing an intelligent early-warning system to predict IDH will greatly assist HD staff in setting optimal dialysate and ultrafiltration parameters (Lin et al., 2019). There are a few studies that focus on the IDH prediction model. Nafisi and Shahabi (2018), Solem et al. (2010), and Sandberg et al. (2014), respectively conducted small sample studies and showed that the finger photoplethysmography (PPG) signal helped predict IDH. However, PPG instruments are not available in all primary hospitals. Huang et al. (2020) integrated five machine learning models (least absolute shrinkage and selection operator, extreme gradient boosting, random forest, support vector regression, and multiple linear regression) to predict BP during HD based on the databaseand found previous BP in the last HD session and first BP reading in the current HD session, which were the most correlated parameters. Lin et al. (2018) developed a prediction model using BP and ultrafiltration records of 667 patients for 30 months. Although these database studies had good accuracy, they ignored the seasonal gradient of BP in HD patients (Duranton et al., 2018). In addition, serum protein levels and blood cells are associated with interdialytic BP. Ozen and Cepken (2020) found that white blood cell (WBC) values were significantly higher in patients developing IDH. The difference between post-dialysis protidemia and pre-dialysis protidemia outperformed BNP (B-natriuretic peptide) and ultrafiltration rate as a predictor for the 30-day risk of IDH (Assayag et al., 2020). Nephrologists still seek a simplified and readily available method, especially in the HD setting, when many patients start treatments while waiting for ultrafiltration prescriptions. Under these circumstances, blood routine test is readily accessible, cost-efficient, and can be of immediate use in any scale HD center. In addition, many scholars have used various machine learning methods to conduct research to explore the relationship between multiple factors and a certain thing.

Liu et al. (2022a) proposed a new chaotic simulated annealing overhaul of the MVO (CSAMVO) and successfully established a hybrid model used for disease diagnosis named CSAMVO-KELM. Liu et al. (2022c) proposed an improved new version of SFLA, that includes a dynamic step size modification method utilizing historical data, a specular reflection learning mechanism, and a simulated annealing process that utilizes chaotic map and levy flight. Moreover, the performance advantages of the method for feature selection were successfully validated in 24 UCI data sets. Shi et al. (2022) combined multiple strategies integrated slime mold algorithm (MSSMA) with KELM technology and successfully proposed a predictive model (MSSMA-KELM) that can be used to predict pulmonary hypertension. El-Kenawy et al. (2020) proposed a feature selection algorithm (SFS-Guided WOA) that combined with well-known classifiers (KNN, SVM, etc.) to achieve a more accurate classification prediction of CT images of covid-19 disease. Elminaam et al. (2021) proposed a new method for dimensionality reduction by combining the Marine Predator Algorithm (MPA) with K-NN and achieved predictions for 18 medical datasets in feature selection. Houssein et al. (2021) proposed a BMO-SVM classification prediction model for more accurate microarray gene expression profiling and cancer classification prediction. Le et al. (2021) used a Gray wolf optimizer (GWO) and the adaptive Particle swarm optimizer (APSO) to optimize the Multilayer perceptron (MLP). They proposed a novel wrapper-based feature selection model for the predictive analysis of early onset in diabetic patients. Senthilkumar et al. (2021) proposed a recursive prediction model based on AI techniques for the prediction of cervical cancer incidence, named the ENSemble classification framework (ENSCF).

In addition, Alagarsamy et al. (2021) introduced a technique that embedded the functionary of the Spatially constricted fish swarm optimization (SCFSO) technique and interval type-II fuzzy logic system (IT2FLS) methodologies, which settled the inaccurate forecasting of anomalies found in various topographical places in brain subjects of magnetic resonance imaging (MRI) modality light. Alshwaheen et al. (2021) proposed a new model based on the long short-term memory-recurrence neural network (LSTM-RNN) combined with the modified Genetic algorithm (GA) to predict the morbidity of ICU patients. Adoko et al. (2013) predicted the rockburst intensity based on a fuzzy inference system (FIS) and adaptive neuro-fuzzy inference system (ANFIS), as well as field measurement data. Cacciola et al. (2013) built a fuzzy ellipsoidal system for environmental pollution prediction using fuzzy rules. Liu et al. (2020) proposed an effective intelligent predictive model (COSCA-SVM) for predicting cervical hyperextension injuries by combining a modified Sine cosine algorithm (SCA) with a support vector machine (SVM). Hu et al. (2022b) proposed a feature selection model (HHOSRL-KELM model) by combining the binary Harris hawk optimization (HHO) algorithm with the specular reflection learning and the kernel extreme learning machine (KELM), which was successfully applied to the severity assessment of covid-19 disease. Therefore, it is feasible to develop a new perspective model based on the swarm intelligence optimization algorithms to predict IDH in this study.

In recent years, a large number of researchers have been exploring ways to combine machine learning techniques with medical diagnostics due to the simplicity of operation, speed of convergence, excellent global convergence performance, and parallelizability of swarm intelligence algorithms. And an increasing number of teams are using swarm intelligence optimization algorithms to optimize the performance of classifiers. For example, there are sine cosine algorithm (SCA) (Mirjalili, 2016), moth-flame optimization (MFO) (Mirjalili, 2015), particle swarm optimization (PSO) (Kennedy and Eberhart, 1995), whale optimization algorithm (WOA) (Mirjalili and Lewis, 2016; Mirjalili et al., 2019), bat-inspired algorithm (BA) (Yang, 2010), gray wolf optimization (GWO) (Mirjalili et al., 2014), grasshopper optimization algorithm (GOA) (Saremi et al., 2017), colony predation algorithm (CPA) (Tu et al., 2021b), slime mould algorithm (SMA) (Li et al., 2020b), hunger games search (HGS) (Yang et al., 2021), weighted mean of vectors (INFO) (Ahmadianfar et al., 2022), Harris hawks optimization (HHO) (Heidari et al., 2019b), Runge Kutta optimizer (RUN) (Ahmadianfar et al., 2021), firefly algorithm (FA) (Yang, 2009), ant colony optimization (ACO) (Dorigo, 1992; Dorigo and Caro, 1999), ant colony optimization based on continuous optimization (ACOR) (Socha and Dorigo, 2008) crow search algorithm (CSA) (Askarzadeh, 2016), and so on. These algorithms have also been successfully applied to several fields, such as optimization of machine learning model (Ling Chen et al., 2014), image segmentation (Hussien et al., 2022; Yu et al., 2022b), medical diagnosis (Xia et al., 2022a,b), economic emission dispatch problem (Dong et al., 2021), plant disease recognition (Yu et al., 2022a), scheduling problems (Gao et al., 2020; Han et al., 2021; Wang et al., 2022), practical engineering problems (Chen et al., 2020; Yu et al., 2022c), multi-objective problem (Hua et al., 2021; Deng et al., 2022b), solar cell parameter Identification (Ye et al., 2021), expensive optimization problems (Li et al., 2020a; Wu et al., 2021a), bankruptcy prediction (Cai et al., 2019; Xu et al., 2019), combination optimization problems (Zhao F. et al., 2021), and feature selection (Hu et al., 2021, 2022a). However, with the development of swarm intelligence and the times, some original heuristic algorithms have gradually revealed their weaknesses in problem optimization, mainly including slow convergence speed, poor convergence accuracy, and easily falling into local optimality in certain problems, etc. Therefore, many scholars have conducted relevant research on original metaheuristic algorithms in the hope that the problem-solving ability of metaheuristic algorithms can be improved. For example, there are chaotic BA (CBA) (Adarsh et al., 2016), boosted GWO (OBLGWO) (Heidari et al., 2019a), modified SCA (mSCA) (Qu et al., 2018), hybrid BA (RCBA) (Liang et al., 2018), hybridizing GWO (HGWO) (Zhu et al., 2015), hybrid SCA and PSO (SCAPSO) (Nenavath et al., 2018), BA based on collaborative and dynamic Learning (CDLOBA) (Yong et al., 2018), and GWO based on cellular automata concept (CAGWO) (Lu et al., 2018).

Inspired by the unique feeding behavior of humpback whales, in 2016, Mirjalili and Lewis (2016) successfully proposed an emerging metaheuristic, named WOA, by imitating the foraging behavior of humpback whales in their natural state, which at the time had a strong ability to find optimal solutions. As the field of the application continues to evolve, the WOA algorithm’s ability to find global optimality in new problem optimization is declining and is prone to fall into local optimality. As a result, a wide range of research has been carried out for WOA, and many improved variants of WOA have been proposed. For example, there are chaotic WOA (CWOA) (Patel et al., 2019), improved WOA (IWOA) (Tubishat et al., 2019), enhanced associative learning-based WOA (BMWOA) (Heidari et al., 2020), A-C parametric WOA (ACWOA) (Elhosseini et al., 2019), lévy flight trajectory-based WOA (LWOA) (Ling et al., 2017), improved opposition-based WOA (OBWOA) (Abd Elaziz and Oliva, 2018), and enhanced WOA (EWOA) (Tu et al., 2021a). Also, many optimized variants of WOA that were proposed by relevant research scholars have been applied to the corresponding areas where they are suitable. For example, Adam et al. (2020) proposed the binary WOA (bWOA) to solve user-base station (BS) association and sub-channel assignment problems. Cao et al. (2021) proposed a hybrid genetic WOA (HGWOA) that optimizes purchased equipment’s production planning and maintenance processes. El-Kenawy et al. (2020) proposed a stochastic fractal search (SFS)-based guided WOA (SFS-Guided WOA) and performed feature classification balancing experiments on it based on COVID-19 images to achieve high accuracy classification prediction of COVID-19 diseases. Ghoneim et al. (2021) proposed an adaptive dynamic polar rose guided WOA (AD-PRS-Guided WOA), which improved the parameters of the classification technique in order to improve the diagnostic accuracy of transformers. Revathi et al. (2020) proposed a genetic WOA by combining a Genetic algorithm (GA) and WOA that successfully overcame the data perturbation problem in cloud computing. Zhao et al. (2022) proposed a hybrid improved WOA and PSO (IWOA-PSO) method and successfully applied it to optimize time jitter path planning to reduce vibration in tandem manipulators and improve robot efficiency.

In the current study, to make WOA better overcome the poor convergence accuracy, easily falling into local optimality and weak stability of WOA for clinical classification prediction, this paper proposes a novel and more excellent variant (COWOA) of WOA for the first time, which introduces the orthogonal learning mechanism and the covariance matrix strategy into the original method to improve its optimization-seeking performance. In COWOA, the orthogonal learning mechanism is first applied to the first half of WOA to increase the population diversity, which is beneficial to the traversal range of the whale population in the solution space and ultimately improves the search ability of the population at that stage. Then, the covariance matrix is applied to the second half of WOA to increase the possibility of each agent jumping out of the local optimum. Eventually, the ability to escape from the local optimum and the convergence accuracy are successfully improved by this method. Finally, the ability of WOA to explore and exploit the global optimum is greatly enhanced by the dual mechanisms. In the process of the COWOA proposal, this paper set up inter-mechanism comparison experiments to verify the algorithm performance of COWOA based on 30 benchmark test functions in IEEE CEC2014. To further enhance the persuasiveness of the COWOA, the paper also compared the COWOA with seven WOA variants, nine original algorithms, and eight optimization variants of other algorithms. Then, this paper combines the binary COWOA (bCOWOA) with the KELM to propose a prediction model for clinical diagnosis and prediction, named the bCOWOA-KELM model. To validate the classification performance of the bCOWOA-KELM model, this paper conducted comparative experiments of the proposed method and other well-known methods based on six public datasets. In addition, to further illustrate the superiority of the bCOWOA-KELM model. a series of classification prediction experiments based on current hospital collected datasets were conducted, including comparison experiments of the different combinations of bCOWOA and six classification methods, comparison experiments of the bCOWOA-KELM model with five well-known classifiers and comparison experiments based on the swarm intelligence optimization algorithm. Moreover, the superiority of the bCOWOA-KELM model was analyzed by four evaluation metrics, including Accuracy, Specificity, Precision, and F-measure. Finally, this paper concluded with a detailed medical discussion of the critical characteristics derived from the experimental results. The main contributions of this study are summarized below.

(1) A higher performance optimization algorithm based on the WOA is proposed, namely COWOA.
(2) A discrete binary algorithm based on the improved COWOA is proposed, named bCOWOA.
(3) A classification prediction model based on bCOWOA and the KELM is proposed, named the bCOWOA-KELM model.
(4) The bCOWOA-KELM model is successfully applied to the classification prediction of IDH.

The rest of the paper is structured as follows. In section “An overview of whale optimization algorithm,” the WOA is introduced, and its basic principles are described. In section “The proposed COWOA,” the improvement process of the COWOA is presented. Section “The proposed bCOWOA-KELM model” shows the proposed process of bCOWOA-KELM. In section “Experiments results and analysis,” we set up comparative experiments to verify the performance of the COWOA and the bCOWOA-KELM model. Section “Discussion” the experimental results and the clinical application of bCOWOA-KELM. Finally, this paper concludes the whole paper and points out future research directions in section “Conclusion and future works.”

## An overview of whale optimization algorithm

The WOA mimics the collaborative behavior of humpback whales during hunting in its search for optimal solutions by driving prey and enclosing them. During the whale’s search and catching of prey, the researchers highlight three key phases: the prey encirclement phase, the bubble net attack phase, and the prey finding phase.

In the surrounding prey phase, other search agents try to perform position updates toward the current optimal position to close to the prey. The behavior is represented by Eq. (2).


(1) $$D=\left|C\cdot X^{*}\,\left(t\right)-X\,\left(t\right)\right|$$

(2) $$X\,\left(t+1\right)=X^{*}\,\left(t\right)-A\cdot D$$

where X* denotes the optimal search agent explored so far. *t* is the number of iterations of the current population update. *D* indicates the distance with the weight between the current best whale position and the current whale position. *A* and *C* are the control parameters of the formula, expressed as in Eq. (3) and Eq. (4).


(3) $$A=2\,a_{1}\cdot r-a_{1}$$

(4) C=2⋅r

(5) $$a_{1}=2-\frac{2\times F\,E\,s}{M\,a\,x\,F\,E\,s}$$

where *r* denotes a random number in the interval [0,1] and *a*_1_ decreases gradually from 2 to 0 as the number of evaluations increases in each iteration. *FEs* is the current number of evaluations, and *MaxFEs* is the maximum number of evaluations.

In the bubble net attack phase, also known as the exploitation phase of the WOA, a total of two whale bubble behaviors are involved, including the enveloping contracting behavior and the spiral updating position. It finds the optimum within a specific range by mimicking how humpback whales attack their prey. When |*A*| < 1, the whales develop a constricting envelope around the prey in the enveloping contracting phase, the essence of the principle is the same as the behavior in the enveloping prey phase, as shown in Eq. (6).


(6) $$X\,\left(t+1\right)=X^{*}\,\left(t\right)-A\cdot D$$

The distance among the whale location and the food location is first computed in the spiral improvement was made, and then a spiral equation is established between the whale and target positions to simulate the spiral motion of the humpback whale, as illustrated in Eq. (7) and Eq. (8).


(7) $$D^{\prime }=\left|X^{*}\,\left(t\right)-X\,\left(t\right)\right|$$

(8) $$X\,\left(t+1\right)=D^{\prime }\cdot e^{b\,l}\cdot \cos\left(2\,\pi \,l\right)+X^{*}\,\left(t\right)$$

where *D*^′^ indicates the distance between the current best and current whale positions. *b* is a constant that can determine the shape of the logarithmic spiral in the mathematical model, and it is set to 1 in the experiment. *l* is a random number between the interval [−2,1] that is used to control the shape of the spiral when the whale attacks the target, as shown in Eq. (9).


(9) $$l=\left(a_{2}-1\right)\cdot r\,a\,n\,d+1$$

(10) $$a_{2}=-1-\frac{F\,E\,s}{M\,a\,x\,F\,E\,s}$$

where *rand* is a random number taking values in the interval [0,1], and *a*_2_ decreases linearly with the number of evaluations in [−2, −1].

In order to ensure the good performance of the whale algorithm, the two update mechanisms are balanced and controlled in the actual model by artificially introducing a random parameter p on the interval [0,1] so that both location update strategies have a 50% probability of being executed. In summary, the complete development model for the attack phase of the bubble network is shown in Eq. (11).


(11) $$X\,\left(t+1\right)=\left\{ \begin{array}{c} X^{*}\,\left(t\right)-A\cdot D,i\,f\,p<0.5\\ D^{\prime }\cdot e^{b\,l}\cdot \cos\left(2\,\pi \,\text{r}\right)+X^{*}\,\left(t\right),i\,f\,p\geq 0.5 \end{array}\right.$$

In the prey finding phase, also known as the exploration phase of the WOA model. In this process, the whale’s position update approach is designed by referring to the position of an arbitrarily selected individual in the whale population. Furthermore, the introduction of random whale individuals increases the diversity of individuals in the population to a certain extent, giving the whales the possibility to jump out of the local optimum to find the optimum. At the same time, parameter *A* is introduced into the process control phase, whose absolute magnitude controls the selection of the whale’s position in the optimization phase.

When |*A*| = 1, the whale’s feeding process enters the prey-seeking phase. Based on the working principles described above, the behaviors of the whale searching for prey during this phase can be defined by Eq. (12) and Eq. (13).


(12) $$D^{\prime \prime }=\left|C\cdot \left(t\right)-X\,\left(t\right)\right|$$

(13) $$X\,\left(t+1\right)=X_{r\,a\,n\,d}\,\left(t\right)-A\cdot D^{\prime \prime }$$

where *X*_*rand*_(*t*) indicates the position of a random individual in the current population. *X*(*t*) Indicates the location of individuals in the current population of whales. *D*^″^ denotes the distance of a random individual whale from the current individual whale under the effect of parameter *C*. *C* is a random number on the interval [0,2].

**FIGURE 1:**
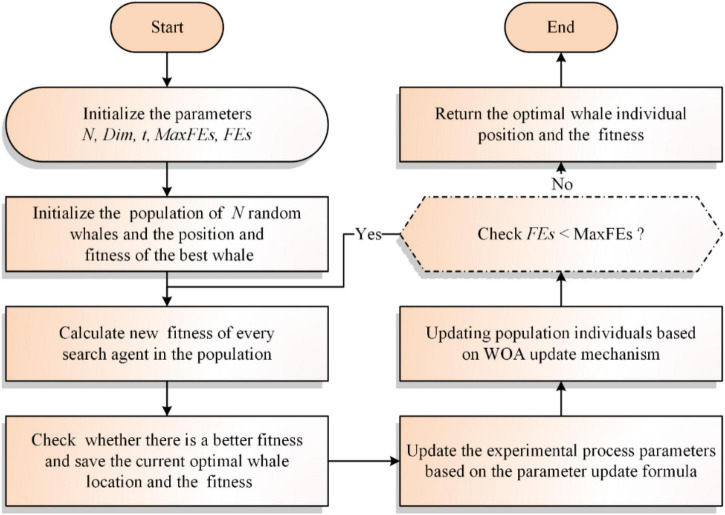
Flowchart of the traditional WOA.

Based on the above, the following paper will give the flowchart and the pseudo-code of the traditional WOA, as shown in Figure 1 and Algorithm 1, respectively.

**Algorithm 1:** Pseudocode for the traditional WOA.

**Input:** The fitness function *F*(*x*), maximum evaluation number (*MaxFEs*), population size (*N*), dimension (*dim*) **Output:** The best Whale (*Leader*_*pos*) Initialize a population of random whales *X* Initialize position vector and score for the leader: *Leader*_*pos*,*Leader*_*score* Initialize the parameters: *FEs, t;* **While** (*FEs* < *MaxFEs*) **For** *i* = 1: size(*X*,1) Return back the search agents that go beyond the boundaries of the search space Calculate objective function for each search agent *FEs* = *FEs* + 1 Update *Leader*_*pos* **End for** **For** *i* = 1: size(*X*,1) Updates the parameters of WOA Position update of population individuals using the population update mechanism of WOA **End for** *t*=*t*+1 **End while** **Return** *Leader*_*pos*

In summary, it is easy to find that the complexity of the WOA is mainly determined by the initializations, updating the population position, updating the weights, and the fitness value calculation. As the time spent by the algorithm is closely related to the specific problem to be solved, the following analysis will focus on the complexity of the WOA in the following aspects, mainly including initializing the population O(N*D), updating the population position *O*(N*D*T), updating the weights *O*(N*T) and calculating the fitness value O(N*T). Therefore, the time complexity of the WOA can be derived as O(((2 + *D*)*T + *D*)*N) by combining the above time complexity analysis. *T* denotes the maximum number of evaluations and can be derived from the maximum number of iterations. *N* denotes the number of individuals in the whale population, and *D* denotes the number of dimensions of the individual whales.

## The proposed COWOA

WOA is one of the more popular population-based metaheuristic optimization algorithms that has been used to solve continuity problems and has achieved significant results in real-world problems. However, the convergence accuracy and convergence speed of the original WOA are not satisfactory, and it tends to fall into local optima. Due to the above two shortcomings of the original WOA, this paper applies the covariance matrix strategy (CMS) and orthogonal learning mechanism (OLM) to WOA, which not only improves the convergence speed and convergence accuracy of the WOA but also enhances its ability to escape from local optima. In this section, we will go into more detail about the optimization process of the COWOA and the two optimization methods of CMS and OLM.

### Covariance matrix strategy

In the original WOA, its guidance of individuals of the whale population in the search for the best focuses on the local area near the best individuals. However, it ignores the possibility of whales finding the best solution near those random individuals, resulting in the original WOA being very susceptible to falling into local optimization. Similarly, when the stochastic parameter *p* < 0.5, the attenuation parameter *a*_1_ controls the exploration and exploitation of the whale population by influencing the absolute value of the key factor *A*. If |*A*| = 1, the algorithm enters the exploration phase. However, as the number of evaluations increases, *a*_1_ decreases linearly from 2 to 0, while the absolute value of |*A*|also gradually decays non-linearly and randomly from 2 to 0. After the number of evaluations increases to a certain level, there is no longer a possibility that |*A*| is greater than 1, so the original WOA cannot find the global optimum at the late stage of each exploration phase. Therefore, this study addresses these shortcomings by introducing a covariance matrix strategy (CMS) (Beyer and Schwefel, 2002; Hu et al., 2021) into the original WOA, allowing WOA to escape from local optima. It works in three main phases, including the sampling phase, the selection and reorganization phase, and the location update phase. Each of them will be described below.

In the sampling phase, the CMS selects a random individual in the whale population and then uses a normal distribution to generate a new population in its vicinity centered on that individual. The process works as shown in Eq. (14).


(14) X⁢(t+1)∼m⁢(t)+σ⁢(t)*N⁢(0,C⁢(t))

where *X*(*t* + 1) denotes the new population generated based on the random solution, and *t* denotes the number of iterations of the population in the evolutionary process. *m* denotes the random solution selected in the whale population during the iteration and is the central individual that generates the next generation population. σ represents the step size of each move. *N* denotes the multinomial normal distribution, and *C* denotes the covariance matrix applied in this operation, as shown in Eq. (16).

In the selection and recombination phase, some representative individuals will be selected from the optimal set of the best individuals obtained after each update and the selected individuals will be recombined to generate a subpopulation relative to the overall population. The formula is shown in Eq. (15).


(15) $$m\,\left(t+1\right)=\sum_{i=1}^{\mu }\omega_{i}*X_{i}\,\left(t+1\right)$$

where *m*(*t* + 1) denotes the central individual of the new population generated for the next iteration, whose position is progressively closer to the optimal solution of the population. *X_i_* denotes the *i*th population individual selected during the iteration. μ denotes the size of the subpopulation. ω_i_denotes the adaptive weights of the corresponding population individuals, and ω_1_ + ω_1_ + ω_3_ + ⋯ + ω_μ_=1.

In the position update phase, this process involves two main update methods, named the *Rank*−*u*−*update* update model and the *Rank*−1−*update* update model, respectively, which guide the individuals of the entire population in the global level search for superiority by updating the covariance matrix of the population. As shown, respectively, in Eq. (16), Eq. (17) and Eq. (18).


(16) $$C\,\left(t+1\right)=\left(1-c_{1}-c_{\mu }\right)\cdot C\,\left(t\right)+R_{1}+R_{u}$$

(17) $$R_{1}=c_{1}\cdot P_{c}\,\left(t+1\right)\cdot {\left(P_{c}\,\left(t+1\right)\right)}^{T}$$

(18) $$R_{u}=c_{\mu }\cdot \sum_{i=1}^{\mu }\omega_{i}\cdot Y_{i}\,\left(t+1\right)\cdot {\left(Y_{i}\,\left(t+1\right)\right)}^{T}$$

(19) $$Y_{i}\,\left(t+1\right)=\frac{\left(X_{i}\,\left(t+1\right)-m\,\left(t\right)\right)}{\sigma \,\left(t\right)}$$

In the above equation, *R*_1_ denotes the *Rank*−1−*update* update mode, *R_u_* denotes the *Rank*−*u*−*update* update mode, and *c*_1_ and *c*_μ_ denote the learning rates of the two update modes, respectively, which are calculated as shown in Eq. (20) and Eq. (21).


(20) $$c_{1}=\frac{2}{D^{2}}$$

(21) $$c_{\mu }\approx \text{min}\,\left(\frac{\mu_{e\,f\,f}}{D^{2}},1-c_{1}\right)$$

where μ_*eff*_ represents a choice set subject to variance, whose mathematical model is shown in Eq. (22)


(22) μe⁢f⁢f=(∑i=1μωi2)-1

During the work of the mechanism, *P_c_* represents how the matrix evolves when the CMS mechanism functions in the search for an advantage, and its update process is shown in Eq. (23).


(23) $$P_{c}\,\left(t+1\right)=\left(1-c_{1}\right)*P_{c}\,\left(t\right)+\sqrt{c_{c}*\left(2-c_{c}\right)*\mu_{e\,f\,f}}\,\left[\frac{m\,\left(t+1\right)-m\,\left(t\right)}{\sigma \,\left(t\right)}\right]$$

In the above equation, *c_c_* denotes the learning rate of *P_c_*; σ is the step parameter in matrix evolution. The initial value of σ is *S*_*best*_/*S* in which *S*_*best*_ is the variance of the global best individual in each dimension relative to the population mean position and *S* is the sum of the variances of each individual in the population in each dimension relative to the population mean position. Its updating process is shown in Eq. (24).


(24) $$\sigma \,\left(t+1\right)=e\,x\,p\,\left(\frac{c_{\sigma }}{d_{\sigma }}\,\left(\frac{||P_{\sigma }\,\left(t+1\right)||}{E\,||N\,\left(0,I\right)||}-1\right)\right)*\sigma \,\left(t\right)$$

where *E*(⋅) is the mathematical expectation function; *I* is a unit matrix used to calculate the step size. *c*_σ_ is the learning rate of σ; *d*_σ_ is the way of the step size that is updated for the damping coefficient. The initial value of *P*_σ_ is equal to 0 and is an evolutionary way of the step size, whose mathematical model is shown in Eq. (25).


(25) $$P_{\sigma }\,\left(t+1\right)=\left(1-c_{c}\right)*P_{\sigma }\,\left(t\right)+\sqrt{c_{c}*\left(2-c_{c}\right)*\mu_{e\,f\,f}}*C\,{\left(t\right)}^{-\frac{1}{2}}*\left[\frac{m\,\left(t+1\right)-m\,\left(t\right)}{\sigma \,\left(t\right)}\right]$$

### Orthogonal learning mechanism

Under the experimental conditions set by the original WOA, the optimal whale search agent in the population was prone to fall into a local optimum (LO) in guiding other group members in the process of finding and apprehending prey, which greatly affected the ability of the whale population to explore and exploit the global optimum (GO). In this paper, an orthogonal learning mechanism (OLM)(Kadavy et al., 2020; Hu et al., 2021) is used to guide the population in a direction closer to the optimal solution by constructing a bootstrap vector after the original whale optimization mechanism, which to some extent improves the convergence speed and the ability of the WOA to explore the optimal solution in the early stages throughout the experiment. The OLM utilized in this experiment is described in the section below.

First, we will locate a guided individual. To better apply the advantages of OLM to the renewal process of whale populations, this study used three randomly selected whale individuals from the original whale population to locate a theoretically relatively better superior whale for the OLM to update the whale population. The expression is shown in Eq. (26).


(26) $$X_{l\,e\,a\,d\,e\,r}=X_{k_{1}}+r\,a\,n\,d\,\left(1,d\,i\,m\right)\cdot \left(X_{k_{2}}-X_{k_{3}}\right)$$

where *X*_*leader*_ represents the guide whale positioned by random individuals *X_k_*_1_, *X_k_*_2_ and *X_k_*_3_ represent the three selected random whales, respectively.

Then, this study introduces the OLM to the WOA and combines it with the acquisition of individual guided whales for guided updating of whale populations. At the same time, to better exploit the advantages of the OLM for exploring whale populations, this study carried out random grouping and hierarchical construction of whale populations and individuals, respectively. We will describe the OLM below; the details can be found in Ref. (Hu et al., 2021).

In order to make full use of each dimension in the individual whale and to better exploit its strengths, this experiment constructed *Q* levels for each dimension. The working model is shown in Eq. (27).


(27) $$L\,e\,v\,e\,l_{q}=X_{i,d}+\frac{q-1}{Q-1}\,\left(X_{j,d}-X_{i,d}\right),q=1,2,\ldots ,Q$$

where *q* denotes the level at which the corresponding dimension is located in the hierarchy construction process.

In each round of experiments, we will obtain the corresponding candidate solutions and then compare each candidate solution’s evaluated values, allowing us to select the best experimental combination solution among many candidates as the current best prediction solution. As shown in Eq. (28).


(28) $${△}_{i,j}=\left(\frac{Q}{M}\right)\,\sum_{Z_{i,j}}f\,i\,t_{i}$$

where *fit*_*i*_denotes the evaluation value corresponding to each orthogonal combination solution generated by OLM; △_*i*,*j*_ is the average evaluation value obtained for each influence factor at each level; *Z* denotes the prediction solution obtained; *M* denotes the number of prediction solutions. Finally, we select the experimental combination solution having the lowest average evaluation value as the best prediction solution by comparing the evaluation value of each candidate solution at different dimensions and levels.

### Implementation of COWOA

**FIGURE 2:**
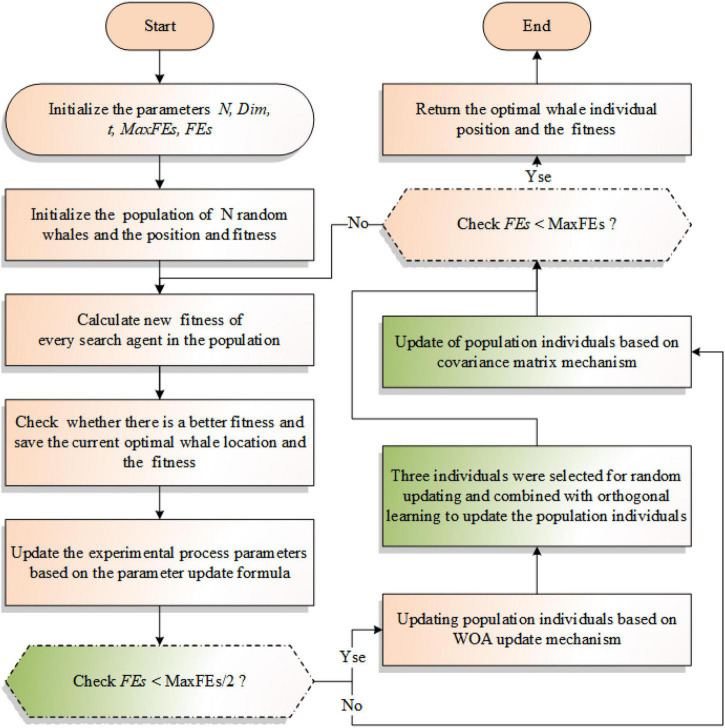
Flowchart of the COWOA.

In this section, the optimization process of COWOA is given based on the above two optimization strategies for the first time. As shown in Algorithm 2, this table describes the overall framework of the COWOA proposed in this paper with pseudo-code. As shown in Figure 2, this chart shows the overall workflow of the COWOA with flowcharts. The COWOA proposed in this paper largely compensates for the shortcomings of exploring and exploiting better solutions for the original WOA. In the first half of the whole experimental process, the exploration ability of the original algorithm is increased by introducing the OLM, which improves the convergence ability of WOA in the early part of the experiment to a certain extent. In the second half of the experimental process, the CMS was introduced to make the original WOA more likely to jump out of the local optimum, greatly improving the population’s search ability and convergence accuracy.

Based on the improvement process and overall workflow of the COWOA, we can find that the initializations mainly determine the complexity of the COWOA, population position update, fitness value calculation, sorting, and the introduction of the OLM and the CMS in WOA together. As the time spent by the algorithm is closely related to the specific problem to be solved, the following analysis will focus on the complexity of COWOA in the following aspects, mainly including initializing the population O(N*D), updating the weights O(N*T), sorting O(N*logN*T), whale position update O(N*D*T/2), CMS update O(N*T/2) and OLM update O(N*(M*D + M*K)*T*/2). Therefore, the time complexity of the COWOA can be derived as O(N*T*(*logN* + (M*D + M*K) + 2) + N*D) by combining the above time complexity analysis. *T* denotes the maximum number of evaluations and can be derived from the maximum number of iterations. *N* denotes the number of individuals in the whale population, and *D* denotes the number of dimensions of the individual whales.

## The proposed bCOWOA-KELM model

### Binary transformation method

It is well known that the WOA is an excellent algorithm proposed for solving continuous problems. Similarly, the COWOA, an improved variant of the WOA proposed in this paper, is also oriented toward continuous problems. However, the core experiments in this paper require a discrete classification technique for feature selection, so the COWOA cannot be applied directly to the feature selection experiments. Therefore, a discrete binary version of the COWOA is proposed, named bCOWOA. The discrete process of the COWOA is described below.

**Algorithm 2:** The pseudocode for the COWOA.

**Input:** The fitness function *F*(x), maximum evaluation number (*MaxFEs*), population size (*N*), dimension (*dim*) **Output:** The best Whale (*Leader*_*pos*) Initialize a population of random whales *X* Initialize position vector and score for the leader: *Leader*_*pos*, *Leader*_*score* Initialize the parameters *FEs, t, Q, F,* *flag, alpha_no* **While** (*FEs* < *MaxFEs*) **For** *i* = 1: size(*X*,1) Return back the search agents that go beyond the boundaries of the search space Calculate the objective function for each search agent *FEs* = *FEs* + 1 Update *Leader*_*pos* Update parameters: *flag, alpha_no* **End for** **If (***FEs* < *MaxFEs*/2) Updating the optimization parameters of the WOA **For** *i* = 1: size(*X*,1) Updating the optimization parameters of the WOA Updating individual whale populations based on the original WOA update mechanism and calculate the assessment value of each individual Locating an individual based on three random individuals Obtain variant individuals using the OLM **End for** **Else** Updating the population of individuals using the CMS **End if** *t*=*t+* 1 **End while** **Return** *Leader*_*pos*

1) Based on the knowledge of discretization techniques, we can quickly determine that the solution domain of a discretization problem is [0,1].
2) As shown in Eq. (29), the bCOWOA is required to convert the searched solution to 0 or 1 by means of the S-shaped transformation function during the experiment.

(29) $$X_{d}\,\left(t+1\right)=\left\{ \begin{array}{c} 1,s\,i\,g\,m\,o\,i\,d\,\left(X_{d}\,\left(t\right)\right)\geq r\\ 0,o\,t\,h\,e\,r\,w\,i\,s\,e \end{array}\right.$$

where *r* is a random number in the interval [0,1]. *X*_*d*_(*t* + 1)denotes a new solution obtained after the binary solution update. 1 indicates that the feature is selected, and 0 indicates that the feature is not selected. And the *sigmoid*(⋅) denotes the S-type transformation function used for the *X*_*d*_(*t*)position update, as shown in Eq. (30).


(30) $$s\,i\,g\,m\,o\,i\,d\,\left(x\right)=\frac{1}{1+e^{-x/3}}$$

where *x* denotes the solution generated during the process of the COWOA.

### Kernel extreme learning machine

The KELM (Tian et al., 2019; Zhang et al., 2020; Zou et al., 2020; Chen H. et al., 2021; Wang and Wang, 2021) is an improved technology based on the Extreme Learning Machine (ELM) combined with a kernel function, which improves the predictive performance of the model while retaining the benefits of the ELM and is a single hidden feedforward neural network with a three-layer independent layer structure, including the input layer, the output layer, and the implicit layer. For a training set with *N* samples: *S*=(*x*_*j*_,*t*_*j*_) ∈ *R*_*n*_×*R*_*m*_, its target learning function model *F*(*x*) can be expressed in Eq. (31).


(31) $$F\,\left(x_{i}\right)=\sum_{i=1}^{L}\beta_{i}\,f\,\left(\omega_{i}\,x_{j}+b_{i}\right)=t_{j},j=1,2,3,\ldots ,N$$

where *x_j_* denotes the *j*th input vector. ω_*i*_ denotes the *i*th random input weight of the input vector. β denotes the *i*th output weight. *f*(ω_*i*_*x*_*j*_ + *b*_*i*_)denotes the activation function of the model. *t_j_* denotes the corresponding output expectation. Following the requirement of scientific research on the principle of simplicity and rigorousness of formulas, Eq. (31) can be rewritten as Eq. (32).


(32) $$T_{N}=H_{N}\,\beta_{N}$$

In the above equation, *T*_*N*_ = [*t*_1_,*t*_2_,*t*_3_,…,*t*_*N*_]^*T*^, β_*N*_ = [β_1_,β_2_,β_3_,…,β_*N*_]^*T*^, *H_N_* is a pre-feedback network matrix consisting of *Nf(⋅). According to the above equation, the functional model of the output weights can be represented by Eq. (33).


(33) $$\beta_{N}=H_{N}^{T}\,{\left(H_{N}\,H_{N}^{T}\right)}^{-1}\,T_{N}$$

A regularization factor C and a unit matrix I can be incorporated to boost the neural network’s reliability, and the least squares result for the final weights is presented in Eq. (34).


(34) $$\beta_{N}=H_{N}^{T}\,{\left(H_{N}\,H_{N}^{T}+\frac{I}{C}\right)}^{-1}\,T_{N}$$

Based on the ELM, the kernel function model is introduced to obtain the KELM whose function model is shown in Eq. (35).


(35) $$Y\,\left(x\right)={\left[\begin{array}{c} K\,\left(x,x_{1}\right)\\ \vdots \\ K\,\left(x,x_{N}\right) \end{array}\right]}^{T}\,{\left(\Omega_{E\,L\,M}+\frac{I}{C}\right)}^{-1}\,T$$

(36) $$\Omega_{\text{ELM}}\,\left(i,j\right)=H\,H^{T}=h\,\left(x_{t}\right)*h\,\left(x_{j}\right)=K\,\left(x_{i},x_{j}\right)$$

(37) $$K\,\left(x_{i},x_{j}\right)=e\,x\,p\,\left(-\frac{{||x_{i}-x_{j}||}^{2}}{\gamma^{2}}\right)$$

where Ω_ELM_ denotes a kernel matrix consisting of kernel functions. *K*(*x*_*i*_,*x*_*j*_) denotes the kernel functions introduced in the ELM. where *x_i_* and *x_j_* denote the input vectors of the sample training set and γ is the parameter in the kernel function.

### Implementation of the bCOWOA-KELM model

This section proposes a novel and efficient model based on the bCOWOA and the KELM for feature selection experiments, named the bCOWOA-KELM model. The model is mainly used to select key features from the dataset. The core model construction method is the optimal solution found by the bCOWOA, and then the optimal solution is extracted by the KELM classifier for secondary classification to improve the classification efficiency and accuracy of the model. In this model, we evaluate the quality of the solution vectors obtained by the bCOWOA through Eq. (38) (Chen et al., 2012, 2013; Hu et al., 2022b,c; Yang et al., 2022a), and use this evaluation as the basis for selecting the optimal solution vector, which is also a key step in the whole feature selection experiment.


(38) $$F\,i\,t\,n\,e\,s\,s=\alpha \cdot e\,r\,r\,o\,r+\beta \cdot \frac{\left|R\right|}{\left|D\right|}$$

where *error* is the error rate of the classifier model. *D* is the dimensionality of the dataset and also represents the number of attributes of the datasets. *R*represents the number of attributes in the subset obtained from the experiment. α is a key parameter used to evaluate the classification and represents the weight for calculating the importance of the error rate. β denotes the length of the selected features. In this paper, α = 0.99 and β = 0.01.

**FIGURE 3:**
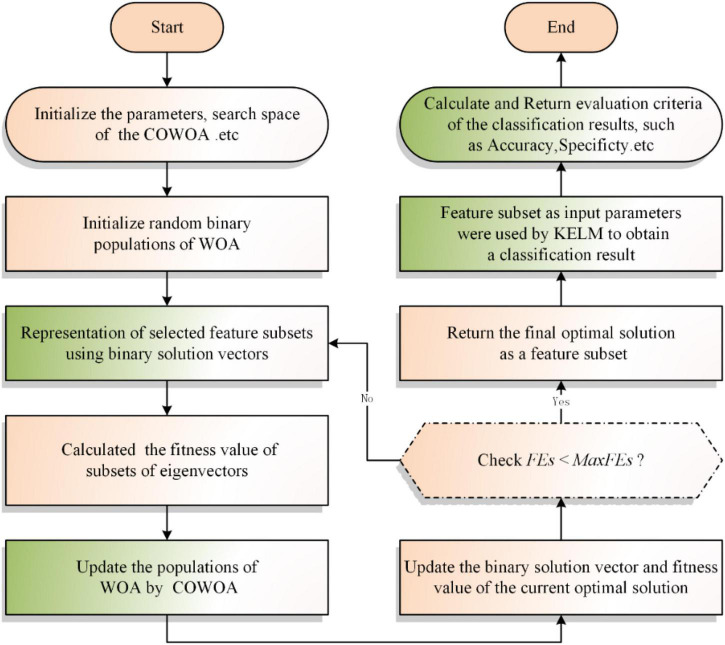
Flowchart of the bCOWOA-KELM model.

In summary, we can obtain the bCOWOA-KELM model by combining the proposed bCOWOA with the KELM in this paper, and its workflow is shown in Figure 3.

## Experiment results and analysis

The purpose of setting up this section is to verify the comprehensive performance of the COWOA and to enhance the persuasiveness of the method proposed in this paper through the analysis results of the experimental data, which mainly include the comparison experiments of the benchmark functions and the classification prediction experiments of the dataset, with two main categories of experimental content. Assessment of computational tasks is a decisive stage that needs benchmarks, available data, and suitable metrics for a valid comparison (Cao Z. et al., 2022; Liu et al., 2022b,d). The evaluation criteria involved include the average value and variance of the relevant experimental data and Accuracy, Specificity, F-measure, and Precision used in the classification prediction experiments. In the benchmark experiment section, the COWOA is compared with the two single-strategy WOA variants and the original WOA, respectively, to demonstrate the better convergence performance of WOA under the dual-strategy effect. To further test the convergence performance of the COWOA, this section also sets up three comparison experiments based on the 30 functions of IEEE CEC2014 so that the COWOA is experimentally compared with seven WOA variants, nine original algorithms, and eight optimized variants of other algorithms, respectively. In the feature selection section, to validate the classification predictive power of the bCOWOA-KELM model and its effectiveness and scalability, the experiments are conducted based on six UCI public datasets and one medical dataset (HD dataset), respectively. Please see below for details of the experiments.

### Benchmark function validation

This section mainly aims to verify the experimental performance of the COWOA proposed in this paper from several aspects, thus providing the basis for the next step of validating the bCOWOA-KELM classification prediction model proposed in this paper.

#### Experiment setup

This section focuses on the basic performance testing of the COWOA proposed in this paper in four aspects, including the comparison between the COWOA optimization mechanisms, the comparison between the COWOA and nine original algorithms, the comparison between the COWOA and seven WOA optimization variants, and the comparison between the COWOA and eight optimization variants of other algorithms. Specific details of the 30 benchmarking functions of IEEE CEC2014 are given in Table 1. The parameters of the involved algorithm are as shown in Table 2 in all function experiments (Table 3).

**TABLE 1:** Description of the 30 benchmark functions.

Class	No.	Functions	${\text{F}}_{i}^{*}={\text{F}}_{i}\,\left({\text{x}}^{*}\right)$
Unimodal functions	1	Rotated high conditioned elliptic function	100
	2	Rotated bent cigar function	200
	3	Rotated discus function	300
Simple multimodal functions	4	Shifted and rotated Rosenbrock’s function	400
	5	Shifted and rotated Ackley’s function	500
	6	Shifted and rotated Weierstrass function	600
	7	Shifted and rotated Griewank’s function	700
	8	Shifted Rastrigin’s Function	800
	9	Shifted and rotated Rastrigin’s Function	900
	10	Shifted Schwefel’s Function	1,000
	11	Shifted and Rotated Schwefel’s Function	1,100
	12	Shifted and rotated Katsuura function	1,200
	13	Shifted and rotated HappyCat function	1,300
	14	Shifted and rotated HGBat function	1,400
	15	Shifted and rotated expanded Griewank’s plus Rosenbrock’s function	1,500
	16	Shifted and rotated expanded Scaffer’s F6 function	1,600
Hybrid functions	17	Hybrid function 1 (*N* = 3)	1,700
	18	Hybrid function 2 (*N* = 3)	1,800
	19	Hybrid function 3 (*N* = 4)	1,900
	20	Hybrid function 4 (*N* = 4)	2,000
	21	Hybrid function 5 (*N* = 5)	2,100
	22	Hybrid function 6 (*N* = 5)	2,200
Composition functions	23	Composition function 1 (*N* = 5)	2,300
	24	Composition function 2 (*N* = 3)	2,400
	25	Composition function 3 (*N* = 3)	2,500
	26	Composition function 4 (*N* = 5)	2,600
	27	Composition function 5 (*N* = 5)	2,700
	28	Composition function 6 (*N* = 5)	2,800
	29	Composition function 7 (*N* = 3)	2,900
	30	Composition function 8 (*N* = 3)	3,000

**TABLE 2:** Description of the parameters in involved algorithms.

Class	Algorithms	Parameters
WOA variants	**COWOA**	**F** = **4**;**Q** = **3**;**a_1_** = [**2**,**0**];**a_2_** = [−**2**,−**1**];**b** = **1**
	ACWOA	*a*_1_ = [2,1];*a*_2_ = [−2,−1];*w* = [0.5,1];*b* = 1
	IWOA	*a*_1_ = [2,0];*a*_2_ = [−2,−1];*b* = 1;*crossover* = 0.1
	BMWOA	*a*_1_ = [2,0];*a*_2_ = [−2,−1];*b* = 1;*bw* = 0.001;*Beta* = 0.1
	LWOA	*a*_1_ = [2,0];*a*_2_ = [−2,−1];*b* = 1
	EWOA	*wMax* = 0.7;*wMin* = 0.2;*a* = [2,0];
	CWOA	*cindex* = 5;*a*_1_ = [2,0];*a*_2_ = [−2,−1];*b* = 1
	OBWOA	*a*_1_ = [2,0];*a*_2_ = [−2,−1];*b* = 1
Basic algorithms	WOA	*a*_1_ = [2,0];*a*_2_ = [−2,−1];*b* = 1
	SCA	*r*1=[2,0]
	GWO	*a* = [2,0]
	MFO	*a* = 2;*b* = 1
	GOA	*cMax* = 0.1;*cMin* = 0.00004
	BA	*Qmin* = 0;*Qmax* = 2
	PSO	*Vmax* = 6;*c*_1_ = 2;*c*_2_ = 2
	CSA	*AP* = 0.1;*f*_1_ = 2
	FA	*alpha* = 0.5;*beta*_*min*_ = 0.2;*gamma* = 1
	ACOR	*k* = 10;*q* = 0.5;*ibslo* = 1
Advanced peers	SCAPSO	*M* = 4;*N* = 9;*c*_1_ = 2;*c*_2_ = 2;*a* = 2
	RCBA	*Qmin* = 0;*Qmax* = 2;*u* = [0,1];*p* = [0,1]
	CBA	*Qmin* = 0;*Qmax* = 2
	HGWO	*beta*_*max*_ = 0.8;*beta*_*min*_ = 0.2;*Cossover* = 0.2;*a* = [2,0]
	OBLGWO	*a*_1_ = [2,0];*a*_2_ = [−2,−1];*b* = 1;*beta* = [2,0]
	mSCA	*JR* = 0.1;a = 2;*r*_1_ = [2,0]
	CDLOBA	*Qmin* = 0;*Qmax* = 2
	CAGWO	*type* = 2;*a* = [2,0]

In addition, when conducting the experimental setup of the baseline functions, we unified some necessary experimental parameters to avoid the influence of internal factors of the experiments, as shown in Table 3.

**TABLE 3:** Parameter setting of the experiment.

Parameter name	Value
The population size	30
Random tests number	30
Objective function dimensions	30
The upper boundary of the search space	100
The lower boundary of the search space	-100
Maximum evaluations number	300,000

For the experimental data, we use the experimentally derived average value (AVG) to reflect the performance of the corresponding algorithm, and the lower the mean value indicates, the more outstanding performance of the algorithm; we use the variance (STG) to reflect the stability of the algorithm, and the lower the variance indicates the relatively more stable performance of the algorithm. Also, in order to further discuss the comprehensive performance of all the algorithms participating in the comparison experiments, the Wilcoxon signed-rank test (García et al., 2010) and Friedman test (García et al., 2010) were also used to analyze the experimental results, and in the paper, respectively, are given in the form of tables and bar charts. In the results of the Wilcoxon signed rank test, “+” in the corresponding table given below indicates that COWOA performs better overall than the other algorithms, “ = ” indicates that it performs almost exactly the same as the other algorithms, and “-” indicates that it performs relatively worse than the other algorithms. Finally, in order to visually discuss the convergence ability of the algorithms and the ability to escape local optima, the partial convergence images of the algorithms are also given in the paper.

We have recommendations in other papers for fair empirical comparison between two or more optimization methods, which demand assigning the same computational resources per approach (Li et al., 2017; Chen J. et al., 2021; Liu K. et al., 2021; Zheng et al., 2022). To ensure the fairness of the external factors, we unified all the experiments based on functions in this section in the same environment, with the parameters of the specific experimental environment, as shown in Table 4.

**TABLE 4:** Description of the experimental environment.

Equipment specifications	Specification parameters
System version	Windows 11 Professional Edition
System type	64-bit operating systems, x64-based processors
Processor	11th Gen Intel(R) Core (TM) i7-11700 @ 2.50GHz 2.50 GHz
RAM on the machine	32.0 GB
Operating equipment	Matlab2021b

#### Impacts of components

This section discusses the proposed process of COWOA, with the main purpose of providing a minimum practical basis for the COWOA proposed in this paper; it also explores the impact of the CMS and the OLM on the WOA during the experimental process and the advantages that the COWOA exhibits in the experiments, such as the superior convergence speed compared to the WOA. Therefore, this subsection sets up a comparison experiment between the COWOA and the CMS-based WOA variant (CMWOA), the OLM-based WOA variant (OWOA), and the original WOA based on the 30 benchmark test functions in IEEE CEC2014.

According to Supplementary Table 1, it is easy to see that among the 30 benchmark function test experiments, the COWOA is relatively more prominent in terms of the number of times it has a smaller mean and variance performance compared to the other three algorithms. Among them, the more the mean value of COWOA, it means that COWOA has more vital exploration and exploitation ability on the optimal problem and also means that it is easier to get a better solution; the more the number of times to get smaller mean value means that COWOA is more adaptable on the optimization problem. In addition, we can see that the variance of the COWOA is relatively small, and the number of performances is relatively high, which indicates that the COWOA proposed in this paper is relatively more stable on the preliminary benchmark function test. Therefore, we can conclude that the comprehensive performance of the COWOA is better when the benchmark functions are optimized using COWOA, CMWOA, OWOA, and WOA, which also tentatively indicates that the COWOA has the prerequisites to be proposed.

To further demonstrate the performance of the COWOA and to enhance the persuasiveness of the COWOA proposed in this paper, two more intuitive statistical methods, the Wilcoxon signed-rank test, and the Freedman test, are used below to analyze and evaluate the experimental data. Table 5 shows the results of the Wilcoxon signed-rank test, where the second column of Table 5 gives the comparative details of the experiments, from which we can see that the COWOA proposed in this paper is superior to the original WOA in as many as 25 out of 30 basis functions, with one having similar performance and only four being relatively poor, which also shows that there is still room for improvement in the COWOA, but does not negate the fact that the COWOA is superior to the original WOA. Similarly, we can see that the COWOA also outperforms the CMWOA and OWOA. Also, the table gives the Wilcoxon ranking, and COWOA is ranked first in the comparison.

**TABLE 5:** Results of Wilcoxon signed-rank test.

Algorithm	+/-/ =	Mean-level	Rank
**COWOA**	**∼**	**1.73**	**1**
CMWOA	11/0/19	2.13	2
OWOA	16/2/12	2.53	3
WOA	25/1/4	3.50	4

Table 6 gives the *P*-values obtained for this experiment based on the Wilcoxon signed-rank test, and the bolded parts in the table represent the results of experiments with *P*-values less than 0.05. By looking at the *P*-values, it can be seen that only a few values are greater than 0.05, which indicates that the COWOA exhibits much better performance than the single mechanism improvement variant and the original WOA in this comparison experiment.

**TABLE 6:** *P*-values of COWOA on the Wilcoxon test.

Functions	CMWOA	OWOA	WOA
	
	*P*-value	*P*-value	*P*-value
F1	3.93334E-01	**1.73440E-06**	**1.73440E-06**
F2	1.00000E+00	**1.73440E-06**	**1.73440E-06**
F3	1.00000E+00	**1.73440E-06**	**1.73440E-06**
F4	2.95878E-01	**1.73440E-06**	**1.73440E-06**
F5	6.26828E-02	**1.73440E-06**	**1.73440E-06**
F6	**3.51524E-06**	7.81264E-01	**3.18168E-06**
F7	**1.95408E-04**	**1.73440E-06**	**1.73440E-06**
F8	**1.73440E-06**	**5.79245E-05**	**1.73440E-06**
F9	**5.21649E-06**	7.03564E-01	**5.30699E-05**
F10	**1.73440E-06**	1.65027E-01	**1.73440E-06**
F11	**6.83586E-03**	**1.83258E-03**	**1.02463E-05**
F12	8.97178E-02	**1.73440E-06**	**1.73440E-06**
F13	1.84622E-01	4.52807E-01	**4.71617E-02**
F14	4.40522E-01	6.58331E-01	2.98944E-01
F15	**2.35342E-06**	**2.70292E-02**	**2.12664E-06**
F16	**4.38962E-03**	3.28571E-01	**2.18267E-02**
F17	1.52861E-01	**1.73440E-06**	**1.73440E-06**
F18	9.42611E-01	**1.73440E-06**	**1.73440E-06**
F19	8.58958E-02	**4.86026E-05**	**1.49356E-05**
F20	5.99936E-01	**1.73440E-06**	**1.73440E-06**
F21	2.80214E-01	**1.73440E-06**	**1.73440E-06**
F22	**3.87230E-02**	1.52861E-01	**4.71617E-02**
F23	1.00000E+00	**1.73440E-06**	**3.11232E-05**
F24	2.53644E-01	**1.11380E-03**	**3.58884E-04**
F25	1.16130E-01	1.58855E-01	4.28430E-01
F26	9.36756E-02	7.52133E-02	3.82034E-01
F27	**1.24526E-02**	5.30440E-01	7.52133E-02
F28	**2.30381E-02**	**1.92092E-06**	**3.06500E-04**
F29	3.82034E-01	2.80214E-01	**1.73440E-06**
F30	1.20445E-01	8.29013E-01	**1.73440E-06**

**FIGURE 4:**
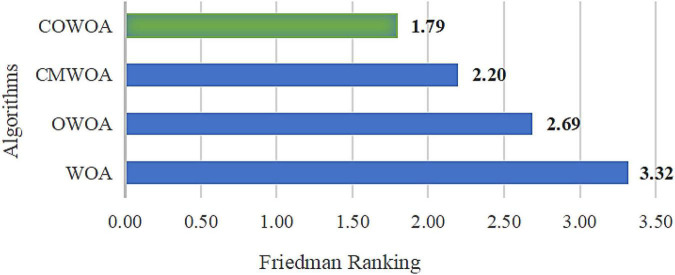
Results of Friedman ranking between the COWOA optimization mechanisms.

Figure 4 Shows the Friedman ranking results of this improvement experiment, from which it can be more intuitively seen that the COWOA can obtain a smaller Friedman average than the other three comparative algorithms in the improvement process under the dual effect of CMS and OLM, which indicates that the COWOA is ranked first in this test method. Therefore, we can tentatively determine that the performance of the COWOA is optimal in this improvement experiment.

**FIGURE 5:**
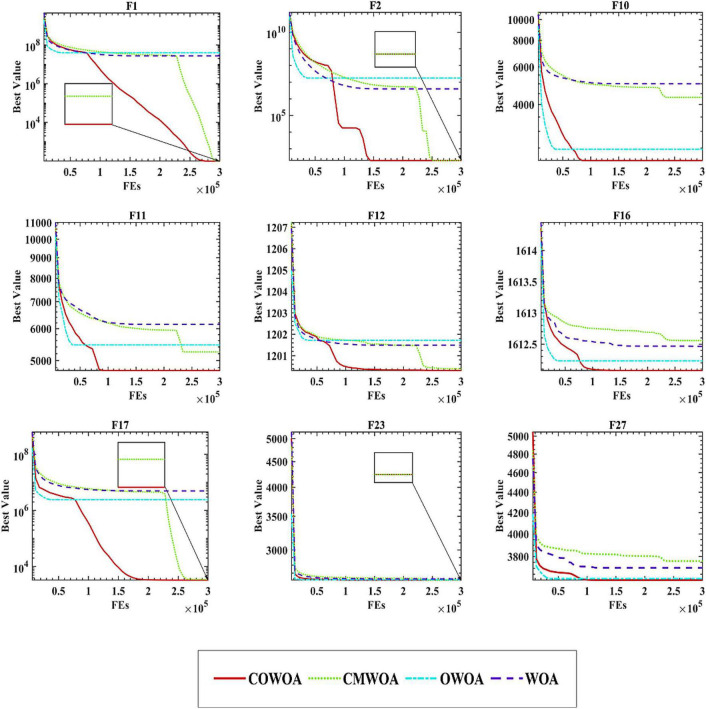
Convergence curves of COWOA performance tests.

Figure 5 gives some of the convergence curves of the four algorithms in the process of finding the optimal solution. The 30 benchmark test functions used in the experiments are divided into four categories: unimodal, simple multimodal, hybrid, and combinatorial functions. Therefore, in order to highlight the performance of the COWOA, we selected the convergence plots so that Figure 5 covers these four categories. It is relatively intuitive to see from the plots that the convergence ability of COWOA on functions F10, F11, and F16 performs very well relative to the other three algorithms. Their convergence curves explore the relatively optimal solutions early in the pre-convergence period, indicating that the COWOA also runs the risk of being unable to escape from the local optimum in some problems. However, the convergence ability it possesses in this experiment is also unmatched by the WOA and cannot be compared. On functions F1, F2, F12, and F17, the shapes of the convergence curves of the COWOA and the CMWOA are extremely similar, but in terms of the final convergence accuracy, except for the equal convergence accuracy on function F2, COWOA is superior; in addition, we can also see that the convergence curve of COWOA reaches a relatively better time earlier than that of CMWOA, which indicates that the COWOA has been strengthened after the introduction of the OLM based on the CMWOA. Not only the convergence accuracy has been improved, but also the convergence speed has been significantly enhanced. The convergence curves of COWOA on functions F1, F2, F11, F12, F16, and F17 all have inflection points in the middle and early stages of the whole experiment, which indicates that the optimization algorithm can escape the local optimum at that stage. In summary, through the comparative experiments and analysis of COWOA with CMWOA, OWOA, and WOA, we can conclude that COWOA is an excellent optimized swarm intelligence algorithm among the improved WOA variants under different combinations of the two optimization methods.

#### Balance analysis experiment

**FIGURE 6:**
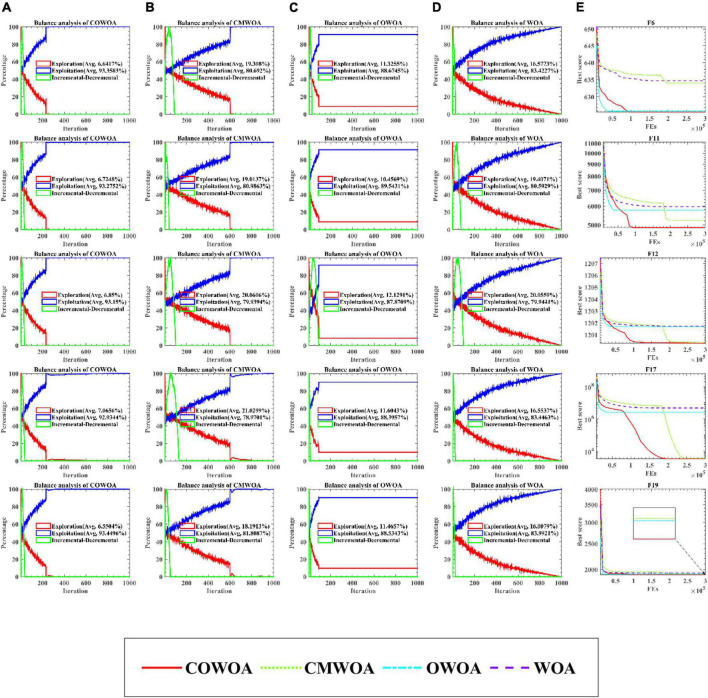
The balance analysis results of COWOA, CMWOA, OWOA, and WOA.

The balance analysis results are given in Figure 6 based on partial benchmark functions. The balance analysis results of COWOA are shown in Figure 6A. The balance analysis results of CMWOA are shown in Figure 6B. The balance analysis results of OWOA are shown in Figure 6C. The balance analysis results of WOA are shown in Figure 6D. Finally, Figure 6E gives the convergence curves obtained by the above four algorithms for the corresponding benchmark function experiments. According to the equilibrium analysis results shown in Figure 6, it can be seen from the functions F6, F12, F17, and F19 that the CMS can enhance the exploration ability of WOA to a certain extent. The OLM improves the development ability of WOA. Combining the results of the equilibrium analysis and the corresponding convergence curves, it can be easily seen that the combination of CMS and OLM can make COWOA reach a better balance point in exploration and exploitation, so that COWOA can obtain better convergence accuracy and convergence speed than CMWOA, OWOA, and WOA in the benchmark function experiments.

#### Comparison with whale optimization algorithm variants

In this subsection, to demonstrate that the COWOA still has outstanding optimization performance in the improved WOA variants and to further enhance the persuasiveness of the COWOA proposed in this paper, seven optimization variants of WOA were purposely selected for comparative experiments and results analysis on 30 benchmark functions. There are CWOA (Patel et al., 2019), IWOA (Tubishat et al., 2019), BMWOA (Heidari et al., 2020), ACWOA (Elhosseini et al., 2019), LWOA (Ling et al., 2017), OBWOA (Abd Elaziz and Oliva, 2018), and EWOA (Tu et al., 2021a). In Supplementary Table 2 shows the AVG and STD of the eight improvement variants of the WOA, including the COWOA, obtained in this experiment. By looking at the table, we can see that the COWOA obtains not only the relatively smallest mean value but also the variance on 13 of the 30 benchmark functions, such as functions F1, F2, F3, F4, F5, F7, F17, F18, F19, F20, F21, F29, and F30. This indicates that the COWOA is able to obtain not only relatively optimal solutions, but also possesses a stability that is difficult to match with the other seven WOA optimization variants. Based on the table of 30 benchmark functions in the experimental setup section, we can find that the 13 listed optimization problems cover the four categories of benchmark functions in the table, which indicates that the COWOA is still more adaptable among the participating WOA variants. In summary, we can conclude that the COWOA is an improved variant of WOA that performs well.

**FIGURE 7:**
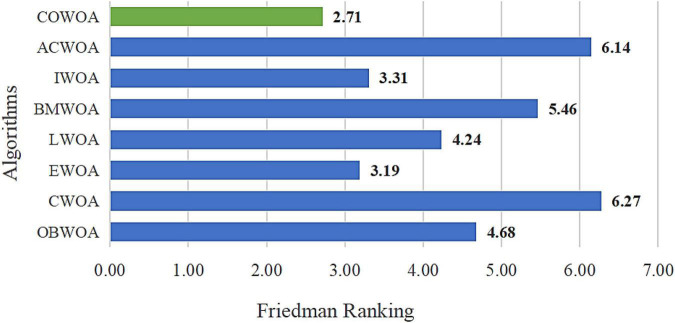
Result of Friedman ranking between the COWOA and the well-known WOA variant algorithms.

In order to further verify the excellent performance of the COWOA, two test results that are statistically significant are presented next, which are the results of the Wilcoxon signed-rank test and the results of Friedman ranking, as shown in Table 7 and Figure 7, respectively. As can be seen from Table 7, the COWOA has a significant average ranking advantage and the final Wilcoxon signed-rank test ranks first among the algorithms involved in the comparison; the second column of Table 7 gives the strengths and weaknesses of the COWOA relative to the other WOA variants in the Wilcoxon signed-rank test. Although the relative strengths and weaknesses are mixed, the final results show that the COWOA is relatively the best.

**TABLE 7:** Results of Wilcoxon signed-rank test.

Algorithm	+/-/ =	Mean-level	Rank
**COWOA**	**∼**	**2.60**	**1**
ACWOA	24/3/3	6.27	7
IWOA	14/7/9	3.10	3
BMWOA	21/5/4	5.20	6
LWOA	20/2/8	4.00	4
EWOA	16/8/6	3.00	2
CWOA	26/0/4	6.93	8
OBWOA	21/5/4	4.87	5

Table 8 gives the *P*-values obtained for this experiment based on the Wilcoxon signed-rank test, and the bolded parts of the table represent the results of experiments with *P*-values greater than 0.05. According to the results in the table, the number of *P*-values greater than 0.05 is a much smaller proportion of the overall results than the proportion less than 0.05, which indicates that the COWOA is superior to the other 7 well-known WOA variants in this evaluation method.

**TABLE 8:** *P*-values of COWOA vs. well-known WOA variants on the Wilcoxon test.

Functions	ACWOA	IWOA	BMWOA	LWOA
	
	*P*-value	*P*-value	*P*-value	*P*-value
F1	1.73440E-06	1.73440E-06	1.73440E-06	1.73440E-06
F2	1.73440E-06	1.73440E-06	1.73440E-06	1.73440E-06
F3	1.73440E-06	1.73440E-06	1.73440E-06	1.73440E-06
F4	1.73440E-06	1.73440E-06	1.73440E-06	1.73440E-06
F5	1.73440E-06	1.73440E-06	1.73440E-06	1.73440E-06
F6	8.46608E-06	1.19734E-03	3.18168E-06	7.73094E-03
F7	1.73440E-06	1.73440E-06	1.73440E-06	1.73440E-06
F8	1.73440E-06	1.73440E-06	1.73440E-06	**1.02011E-01**
F9	1.47728E-04	**8.22065E-02**	4.89690E-04	1.31942E-02
F10	1.73440E-06	3.51524E-06	1.73440E-06	**6.58331E-01**
F11	7.69086E-06	7.27105E-03	1.73440E-06	1.24526E-02
F12	1.73440E-06	**5.19307E-02**	1.73440E-06	5.21649E-06
F13	1.63945E-05	**2.13358E-01**	**1.20445E-01**	**6.88359E-01**
F14	1.92092E-06	**5.71646E-01**	**8.13017E-01**	2.56371E-02
F15	1.92092E-06	2.30381E-02	2.35342E-06	2.05153E-04
F16	**4.77947E-01**	2.41470E-03	**2.89477E-01**	**2.13358E-01**
F17	1.73440E-06	1.73440E-06	1.73440E-06	1.73440E-06
F18	1.73440E-06	1.92092E-06	1.73440E-06	1.73440E-06
F19	1.73440E-06	**5.85712E-01**	1.73440E-06	2.76527E-03
F20	1.73440E-06	7.51366E-05	1.73440E-06	2.12664E-06
F21	1.73440E-06	1.73440E-06	1.73440E-06	1.73440E-06
F22	4.38962E-03	**3.49346E-01**	4.68184E-03	**5.98356E-02**
F23	7.73527E-08	1.73440E-06	1.73440E-06	1.73440E-06
F24	1.73440E-06	1.73440E-06	3.11232E-05	1.97295E-05
F25	2.70159E-05	**5.66635E-01**	2.59671E-05	1.89097E-04
F26	4.44934E-05	**5.57743E-01**	**1.30592E-01**	**7.65519E-01**
F27	**3.70935E-01**	1.48393E-03	1.73440E-06	**2.13358E-01**
F28	**7.52133E-02**	**2.89477E-01**	1.73440E-06	**5.98356E-02**
F29	3.18168E-06	1.73440E-06	1.73440E-06	1.73440E-06
F30	4.72920E-06	1.73440E-06	1.92092E-06	1.73440E-06

**Functions**	**EWOA**		**CWOA**	**OBWOA**
	
	***P*-value**		***P*-value**	***P*-value**

F1	1.73440E-06		1.73440E-06	1.73440E-06
F2	1.73440E-06		1.73440E-06	1.73440E-06
F3	1.73440E-06		1.73440E-06	1.73440E-06
F4	1.73440E-06		1.73440E-06	1.73440E-06
F5	1.73440E-06		1.73440E-06	1.73440E-06
F6	9.84214E-03		1.73440E-06	2.84342E-05
F7	1.73440E-06		1.73440E-06	1.73440E-06
F8	1.73440E-06		1.73440E-06	1.73440E-06
F9	**1.02011E-01**		8.30707E-04	4.89690E-04
F10	1.48393E-03		1.73440E-06	1.73440E-06
F11	2.18267E-02		1.73440E-06	4.72920E-06
F12	4.07023E-02		1.73440E-06	1.73440E-06
F13	**8.22065E-02**		**9.36756E-02**	4.71617E-02
F14	**7.52133E-02**		**1.71376E-01**	**1.20445E-01**
F15	3.11232E-05		1.73440E-06	1.73440E-06
F16	4.11403E-03		2.41470E-03	**9.58990E-01**
F17	1.73440E-06		1.73440E-06	1.73440E-06
F18	8.46608E-06		1.73440E-06	1.73440E-06
F19	**1.84622E-01**		1.73440E-06	1.73440E-06
F20	2.18267E-02		1.73440E-06	1.73440E-06
F21	1.73440E-06		1.73440E-06	1.73440E-06
F22	4.71617E-02		7.73094E-03	**1.35908E-01**
F23	1.73440E-06		3.11232E-05	4.32046E-08
F24	1.97295E-05		2.59671E-05	3.11232E-05
F25	1.59094E-02		**6.56707E-01**	2.70159E-05
F26	**1.58855E-01**		2.25512E-03	1.92092E-06
F27	3.50090E-02		**3.49346E-01**	1.73440E-06
F28	**4.16534E-01**		7.15703E-04	6.83586E-03
F29	1.73440E-06		1.73440E-06	**6.73280E-01**
F30	1.73440E-06		1.73440E-06	2.76527E-03

As seen in Figure 7, the COWOA obtained a Friedman mean of 2.71 and the smallest compared to the seven well-known WOA improvement variants, indicating that the COWOA ranks first under this test method. For the other seven compared algorithms, the mean value obtained by COWOA is 0.48 smaller than the second-ranked EWOA and 3.56 smaller than the poorly ranked CWOA. In summary, we can prove through this experiment that the COWOA is an excellent new variant of WOA improvement.

**FIGURE 8:**
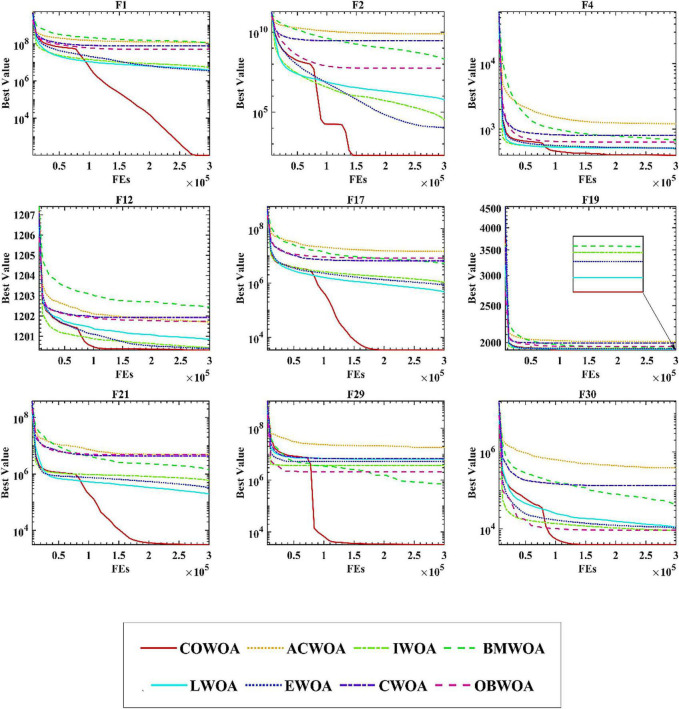
Convergence curves of COWOA with the WOA variants.

To confirm the superiority of the COWOA, we next took the convergence curves obtained throughout the experiment and selected images of the optimization process for nine functions according to the principle of including four classes of benchmark functions, as shown in Figure 8. The convergence curves of the COWOA on the listed functions are significantly better than those of the other seven WOA variants, except for F9, which shows that the COWOA is relatively the strongest in these optimization problems. On functions F1, F2, F12, F17, F21, F29, and F30, the other seven WOA variants’ convergence curves are relatively smooth. In contrast, the convergence curves of the COWOA have one or two inflection points in the middle and early stages of the experiment, which indicates that the COWOA can escape the local optimum early in the process of finding the optimal solution and that the convergence achieved in the end is the accuracy is also relatively optimal. In summary, in the comparative experiments set up in this paper, the convergence ability of the COWOA proposed can achieve relatively better than the seven WOA variants of the algorithm. Therefore, this experiment proves that the COWOA is an excellent WOA variant.

**FIGURE 9:**
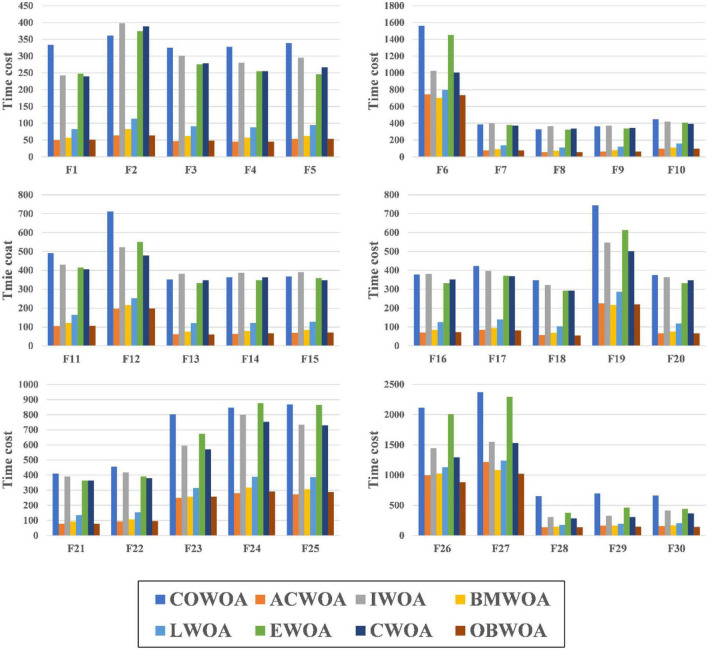
The computational cost of COWOA with the WOA variants.

The results of the experiments that compare the computational costs of COWOA and the 7 WOA variants are shown in Figure 9. To ensure the experiment’s fairness, the experiment uniformly uses the same experimental setup as the benchmark function validation experiments. In addition, the results of this experiment are counted in seconds. As seen in Figure 9, COWOA, IWOA, EWOA, and CWOA have similar time costs in optimizing the 30 basic problems, and all have relatively higher complexity than ACWOA, BMWOA, LWOA, and OBWOA. This is due to the different complexity of the optimization methods introduced in the WOA variants. For COWOA, the CMS and the OLM bring more computational overhead to it. The comprehensive analysis of the experimental results presented in this section concludes that COWOA is computationally acceptable in terms of the time cost spent.

#### Comparison with basic algorithms

In this subsection, we set up a comparison experiment between the COWOA and the well-known original algorithms of today, and there are SCA, MFO, PSO, WOA, BA, GWO, GOA, FA, ACOR, and CSA. The main purpose of this section is to provide a practical basis for the COWOA proposed in this paper and to further demonstrate that the COWOA also has relatively outstanding convergence capabilities among other original algorithms.

Supplementary Table 3 presents the corresponding experimental results in the form of AVG and STG in Supplementary Table. By comparing and looking at the mean values; we can see that the COWOA exhibits the smallest mean values for functions F1, F2, F3, F4, F7, F10, F12, F17, F18, F19, F20, F21, F23, F29, and F30 out of the 30 is benchmark functions. The COWOA excelled in this area with 15 benchmark functions compared to other algorithms, a capability that other algorithms participating in the comparison experiments did not have. In addition, the COWOA showed the smallest variance in functions F1, F2, F3, F4, F7, F12, F17, F18, F20, F21, F23, F29, and F30, indicating that the COWOA is relatively more stable in its optimization of these benchmark functions. In summary, the COWOA is not only highly adaptive to the optimization problem, but also achieves relatively better solutions in comparison with the nine well-known original algorithms; the relatively small variance of the optimal solution for most of the problems demonstrates that the COWOA is more stable most of the time. Therefore, the overall capability of the COWOA is worthy of recognition, and the COWOA is a very good improvement algorithm.

To make the experimental results more scientific, the Wilkerson signed-rank test was used to evaluate the experimental results below, as shown in Table 9. According to Table 9, we can see that the COWOA ranks first in the overall ranking of the comparison experiments in this setup. The two columns Mean-level and Rank give the final ranking data more intuitively. In addition, the second column of the table shows the details of the experimental results based on 30 benchmark functions, from which it is easy to see that the original algorithm performs better on at least 17 optimization problems and at most 28 optimization problems for the different original algorithms. Although the performance on some problems is not as outstanding as the other original algorithms, this does not affect the overall performance of the COWOA, which is relatively the best among them, but only shows that the COWOA still has much room for optimization in the future.

**TABLE 9:** Results of Wilcoxon signed-rank test.

Algorithm	+/-/ =	Mean-level	Rank
**COWOA**	**∼**	**2.90**	**1**
SCA	26/2/2	8.27	9
GWO	18/8/4	5.03	6
MFO	25/2/3	7.10	8
GOA	19/8/3	4.33	4
BA	26/2/2	5.70	7
PSO	22/6/2	4.90	5
CSA	19/4/7	3.80	2
FA	28/1/1	8.83	10
ACOR	17/8/5	4.13	3

Further validation of the Wilcoxon signed-rank test is given in Table 10, where the bold data indicates a *p*-value greater than 0.05. The number of *p*-values greater than 0.05 in the overall results of the COWOA against the 9 well-known original algorithms is very small, which proves that the COWOA still performs very well in comparison with the well-known WOA variants.

**TABLE 10:** *P*-values of COWOA vs. the well-known original algorithms on the Wilcoxon test.

Functions	SCA	GWO	MFO	GOA	BA
	
	*P*-value	*P*-value	*P*-value	*P*-value	*P*-value
F1	1.73440E-06	1.73440E-06	1.73440E-06	1.73440E-06	1.73440E-06
F2	1.73440E-06	1.73440E-06	1.73440E-06	1.73440E-06	1.73440E-06
F3	1.73440E-06	1.73440E-06	1.73440E-06	1.73440E-06	1.73440E-06
F4	1.73440E-06	1.73440E-06	1.73440E-06	1.73440E-06	5.30699E-05
F5	1.73440E-06	1.73440E-06	1.92092E-06	1.73440E-06	1.73440E-06
F6	1.92092E-06	1.73440E-06	**8.22065E-02**	4.72920E-06	2.60333E-06
F7	1.73440E-06	1.73440E-06	2.56308E-06	1.73440E-06	1.73440E-06
F8	1.73440E-06	**3.60039E-01**	1.73440E-06	6.15641E-04	1.73440E-06
F9	1.73440E-06	6.33914E-06	4.19551E-04	1.79885E-05	2.35342E-06
F10	1.73440E-06	5.21649E-06	1.73440E-06	1.73440E-06	1.73440E-06
F11	1.73440E-06	1.35948E-04	1.31942E-02	**6.14315E-01**	1.60464E-04
F12	1.73440E-06	4.11403E-03	6.89229E-05	2.37045E-05	1.73440E-06
F13	1.73440E-06	4.99155E-03	1.73440E-06	7.73094E-03	**1.35908E-01**
F14	1.73440E-06	5.31968E-03	1.73440E-06	**5.98356E-02**	**3.93334E-01**
F15	1.73440E-06	**2.71155E-01**	1.92092E-06	2.35342E-06	**2.53644E-01**
F16	3.88111E-04	5.21649E-06	1.47954E-02	3.60943E-03	2.60333E-06
F17	1.73440E-06	1.73440E-06	1.73440E-06	1.73440E-06	1.73440E-06
F18	1.73440E-06	1.73440E-06	1.73440E-06	1.73440E-06	1.73440E-06
F19	1.73440E-06	2.60333E-06	1.23808E-05	1.56585E-02	4.07151E-05
F20	1.73440E-06	1.73440E-06	1.73440E-06	5.21649E-06	3.51524E-06
F21	1.73440E-06	1.73440E-06	1.73440E-06	1.73440E-06	1.73440E-06
F22	**1.02011E-01**	1.47728E-04	4.68184E-03	4.99155E-03	6.33914E-06
F23	1.73440E-06	1.73440E-06	1.73440E-06	1.73440E-06	1.73440E-06
F24	3.32689E-02	**1.77907E-01**	1.73440E-06	1.92092E-06	1.73440E-06
F25	1.96458E-03	**1.98610E-01**	**3.38856E-01**	5.31968E-03	2.22483E-04
F26	3.58884E-04	4.16912E-03	3.58884E-04	2.58456E-03	**6.28843E-01**
F27	**2.53644E-01**	5.31968E-03	**4.16534E-01**	3.68261E-02	1.70877E-03
F28	2.22483E-04	9.27103E-03	2.76527E-03	**1.35908E-01**	1.36011E-05
F29	1.73440E-06	1.73440E-06	1.73440E-06	1.73440E-06	1.73440E-06
F30	1.73440E-06	1.73440E-06	1.73440E-06	1.73440E-06	1.73440E-06

**Functions**	**PSO**	**CSA**		**FA**	**ACOR**
	
	***P*-value**	***P*-value**		***P*-value**	***P*-value**

F1	1.73440E-06	1.73440E-06		1.73440E-06	1.73440E-06
F2	1.73440E-06	1.73440E-06		1.73440E-06	1.73331E-06
F3	1.73440E-06	1.73440E-06		1.73440E-06	1.73440E-06
F4	1.73440E-06	1.73440E-06		1.73440E-06	1.90777E-06
F5	1.73440E-06	7.69086E-06		1.73440E-06	1.73440E-06
F6	6.83586E-03	1.74228E-04		1.92092E-06	1.73440E-06
F7	1.73440E-06	1.73440E-06		1.73440E-06	1.94383E-04
F8	1.73440E-06	2.12532E-06		1.73440E-06	3.60943E-03
F9	1.36011E-05	**4.16534E-01**		1.73440E-06	2.30381E-02
F10	1.73440E-06	1.73440E-06		1.73440E-06	1.12654E-05
F11	2.87860E-06	**1.15608E-01**		1.73440E-06	**8.61213E-01**
F12	1.73440E-06	2.35342E-06		1.73440E-06	1.73440E-06
F13	3.11232E-05	**6.73280E-01**		1.73440E-06	**6.14315E-01**
F14	2.10526E-03	1.24526E-02		1.73440E-06	1.83258E-03
F15	4.44934E-05	**1.58855E-01**		1.73440E-06	**9.42611E-01**
F16	2.70292E-02	**5.85712E-01**		2.61343E-04	3.40526E-05
F17	1.73440E-06	1.73440E-06		1.73440E-06	1.73440E-06
F18	1.73440E-06	1.25057E-04		1.73440E-06	1.92092E-06
F19	8.91873E-05	1.12654E-05		1.73440E-06	**8.77403E-01**
F20	6.33914E-06	2.84342E-05		1.73440E-06	1.73440E-06
F21	1.73440E-06	1.73440E-06		1.73440E-06	1.73440E-06
F22	**1.30592E-01**	**5.85712E-01**		2.95746E-03	1.48393E-03
F23	1.73440E-06	1.73440E-06		1.73440E-06	1.11234E-06
F24	2.35342E-06	1.12654E-05		1.73440E-06	1.73440E-06
F25	**2.21022E-01**	2.06711E-02		2.35342E-06	1.83258E-03
F26	1.73440E-06	**7.52133E-02**		3.58884E-04	**6.87136E-02**
F27	8.72967E-03	3.06500E-04		4.19551E-04	1.75184E-02
F28	2.35342E-06	1.73440E-06		**7.49871E-01**	2.41180E-04
F29	1.28663E-03	1.73440E-06		1.73440E-06	1.73440E-06
F30	1.73440E-06	1.73440E-06		1.73440E-06	1.73440E-06

**FIGURE 10:**
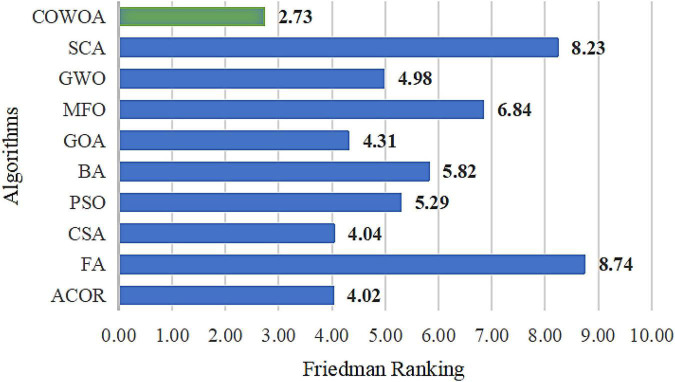
Result of Friedman ranking between the COWOA and the well-known original algorithm.

The results of the Friedman test are given in Figure 10, from which it can be seen that the COWOA has the smallest mean value of the Friedman compared with the nine well-known original algorithms, which indicates that the COWOA is ranked first in this basis function experiment. If the difference between the second-ranked ACOR and the COWOA is defined as △, then the absolute value of △ is greater than 1, |△| > 1. As a result, it can be proven that the COWOA still has an obvious advantage under this evaluation method.

**FIGURE 11:**
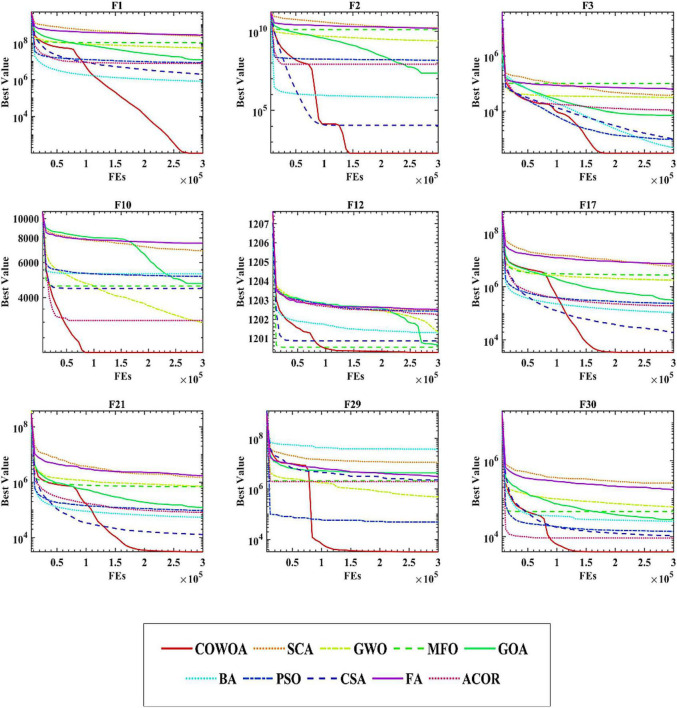
Convergence curves of COWOA with the well-known original algorithms.

To further demonstrate the benefits of COWOA, this subsection also uses the same experimental structure set up in the performance testing section of COWOA to give convergence curves for the entire iterative process and the convergence curves for the nine functions selected by the four classes of benchmark functions, as shown in Figure 11. It can be seen from the convergence plots that the convergence speed of COWOA has an undeniable advantage over the entire iterative process. The ability to develop a global optimum is relatively the best relative to the nine original algorithms. In addition, the convergence curves of the COWOA on functions F1, F2, F17, F21, F29, and F30 all have obvious inflection points, and the inflection points are relatively forward, which indicates that the optimization algorithm has an undeniable ability to escape the local optimum in the early stage of finding the optimal solution, which is a convergence performance that the other nine original algorithms do not have. In summary, through the analysis of the comparative experiments in this setup, we can conclude that the COWOA proposed in this paper is an excellent variant of WOA.

#### Comparison with advanced peers

In this section, we make a comparative experiment and analysis of COWOA with eight optimization variants of other algorithms, which will be the most powerful interpretation of COWOA performance in the experimental part of this paper on basis functions. They are CBA (Adarsh et al., 2016), OBLGWO (Heidari et al., 2019a), mSCA (Qu et al., 2018), RCBA (Liang et al., 2018), HGWO (Zhu et al., 2015), SCAPSO (Nenavath et al., 2018), CDLOB (Yong et al., 2018), and CAGWO (Lu et al., 2018). Supplementary Table 4 shows the AVG and STG of the benchmark experimental results of COWOA against the eight improved algorithms. By comparing and looking at the average values, we find that for most of the benchmark functions, COWOA has the smallest average value. This indicates that the COWOA has a relatively higher quality optimization capability in this experiment. Furthermore, the relatively smallest variance of the optimal solutions obtained is a strong indication of the better stability of the COWOA’s optimization capability on these benchmark functions. Therefore, we can tentatively conclude that the COWOA is a novel and excellent improvement algorithm.

Table 11 shows the final analysis and evaluation details of the Wilkerson signed-rank test, from which it can be seen that the mean of the COWOA has a significant advantage in this set-up of the comparison experiment and is ranked first among the compared algorithms. In addition, the second column of the table gives the degree of superiority of the COWOA compared to the other eight algorithms, from which we can see that the COWOA exhibits relatively more outstanding optimization capabilities for most of the optimization problems, which not only demonstrates the greater performance of the COWOA but also proves that it has better adaptability to many optimization problems.

**TABLE 11:** Results of Wilcoxon signed-rank test.

Algorithm	+/-/ =	Mean-level	rank
**COWOA**	**∼**	**2.37**	**1**
SCAPSO	20/8/2	4.00	2
RCBA	27/0/3	5.13	5
CBA	27/0/3	6.03	8
HGWO	24/3/3	6.67	9
OBLGWO	18/5/7	4.40	3
mSCA	21/5/4	5.53	6
CDLOBA	25/0/5	5.73	7
CAGWO	19/9/2	4.90	4

Table 12 shows the *p*-values of the COWOA for the eight well-known variants, where the bolded data indicate *p*-values greater than 0.05. According to the table, the number of data less than 0.05 occupies the majority of the overall portion, indicating that the COWOA performs better than the well-known variant algorithms.

**TABLE 12:** The *p*-values of COWOA vs. well-known algorithms on the Wilcoxon test.

Functions	SCAPSO	RCBA	CBA	HGWO
	
	*P*-value	*P*-value	*P*-value	*P*-value
F1	1.73440E-06	1.73440E-06	1.73440E-06	1.73440E-06
F2	1.73440E-06	1.73440E-06	1.73440E-06	1.73440E-06
F3	1.73440E-06	1.73440E-06	1.73440E-06	1.73440E-06
F4	1.73440E-06	2.12664E-06	1.73440E-06	1.73440E-06
F5	1.73440E-06	1.73440E-06	3.51524E-06	1.73440E-06
F6	1.25057E-04	2.12664E-06	1.73440E-06	**8.29013E-01**
F7	1.73440E-06	1.73440E-06	2.00130E-05	1.73440E-06
F8	1.73440E-06	1.73440E-06	1.73440E-06	1.73440E-06
F9	1.47728E-04	1.73440E-06	3.88218E-06	2.12664E-06
F10	1.73440E-06	1.73440E-06	1.73440E-06	1.73440E-06
F11	5.28725E-04	3.60943E-03	7.15703E-04	1.92092E-06
F12	1.73440E-06	6.31976E-05	1.92092E-06	1.73440E-06
F13	**1.58855E-01**	**9.75387E-01**	**7.65519E-01**	1.73440E-06
F14	7.51366E-05	**6.58331E-01**	**2.36936E-01**	1.73440E-06
F15	1.47728E-04	**5.70965E-02**	2.16302E-05	1.73440E-06
F16	**1.35908E-01**	2.60333E-06	1.73440E-06	9.27103E-03
F17	1.73440E-06	1.73440E-06	1.73440E-06	1.73440E-06
F18	1.73440E-06	1.73440E-06	1.73440E-06	1.73440E-06
F19	4.28569E-06	6.33914E-06	2.60333E-06	1.73440E-06
F20	6.98378E-06	3.72426E-05	1.73440E-06	1.73440E-06
F21	1.73440E-06	1.73440E-06	1.73440E-06	1.73440E-06
F22	5.30699E-05	1.92092E-06	1.73440E-06	5.30699E-05
F23	4.32046E-08	1.73440E-06	1.73440E-06	1.81225E-06
F24	1.73440E-06	1.73440E-06	1.73440E-06	1.73440E-06
F25	2.70159E-05	4.07151E-05	5.75165E-06	2.70159E-05
F26	2.16302E-05	1.31942E-02	**5.99936E-01**	2.60333E-06
F27	1.73440E-06	2.43075E-02	1.35948E-04	**2.21022E-01**
F28	1.73440E-06	1.23808E-05	4.44934E-05	**2.53644E-01**
F29	3.06500E-04	1.73440E-06	1.73440E-06	1.14992E-04
F30	1.23808E-05	1.73440E-06	1.73440E-06	3.11232E-05

**Functions**	**OBLGWO**	**mSCA**	**CDLOBA**	**CAGWO**
	
	***P*-value**	***P*-value**	***P*-value**	***P*-value**

F1	1.73440E-06	1.73440E-06	1.73440E-06	1.73440E-06
F2	1.73440E-06	1.73440E-06	1.73440E-06	1.73440E-06
F3	1.73440E-06	1.73440E-06	1.73440E-06	1.73440E-06
F4	1.73440E-06	1.73440E-06	2.12664E-06	1.73440E-06
F5	1.73440E-06	1.73440E-06	1.73440E-06	1.73440E-06
F6	6.15641E-04	4.07151E-05	1.73440E-06	1.73440E-06
F7	1.73440E-06	1.73440E-06	5.75165E-06	1.73440E-06
F8	8.46608E-06	3.18168E-06	1.73440E-06	**2.28880E-01**
F9	**4.16534E-01**	**7.34325E-01**	1.73440E-06	3.16034E-02
F10	1.73440E-06	1.73440E-06	1.73440E-06	7.69086E-06
F11	**6.56411E-02**	**7.97098E-01**	3.60943E-03	1.05695E-04
F12	1.73440E-06	2.16302E-05	**9.36756E-02**	1.73440E-06
F13	**5.44006E-01**	1.63945E-05	**3.70935E-01**	1.48393E-03
F14	2.41470E-03	3.51524E-06	**9.26255E-01**	1.74228E-04
F15	2.22483E-04	1.73440E-06	1.73440E-06	3.60943E-03
F16	**1.20445E-01**	3.16176E-03	1.92092E-06	1.83258E-03
F17	1.73440E-06	1.73440E-06	1.73440E-06	1.73440E-06
F18	1.73440E-06	1.73440E-06	1.73440E-06	1.73440E-06
F19	**9.42611E-01**	1.73440E-06	1.73440E-06	1.73440E-06
F20	1.73440E-06	1.73440E-06	1.73440E-06	1.73440E-06
F21	1.73440E-06	1.73440E-06	1.73440E-06	1.73440E-06
F22	**7.97098E-01**	1.17481E-02	2.60333E-06	4.68184E-03
F23	3.11232E-05	1.73440E-06	1.73440E-06	1.73440E-06
F24	1.24526E-02	**7.86467E-02**	1.73440E-06	**8.93644E-01**
F25	2.70159E-05	**3.08615E-01**	1.48393E-03	2.70159E-05
F26	**8.97178E-02**	2.12664E-06	**8.58958E-02**	3.16034E-02
F27	4.28569E-06	2.59671E-05	**3.28571E-01**	1.36011E-05
F28	1.05695E-04	7.71217E-04	3.40526E-05	9.31566E-06
F29	1.73440E-06	1.73440E-06	1.73440E-06	1.73440E-06
F30	1.73440E-06	1.73440E-06	1.73440E-06	1.73440E-06

**FIGURE 12:**
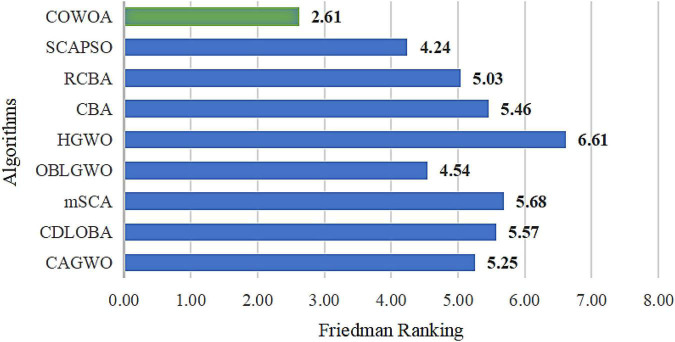
Result of Friedman ranking between the COWOA and the well-known variant algorithms.

Figure 12 shows the result of the Friedman ranking, from which it can be seen that the COWOA has the smallest average, which indicates that it still has a very strong advantage under this testing method. Therefore, COWOA is still a relatively better swarm intelligence optimization algorithm for the experiments in this setup.

**FIGURE 13:**
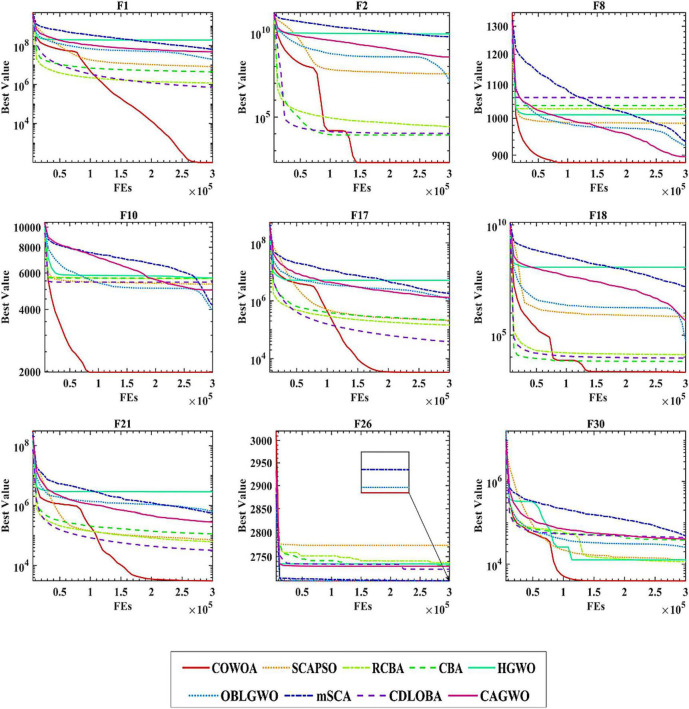
Convergence curves of COWOA with the well-known variant algorithms.

In order to enhance the conviction that the optimization capability of the COWOA is relatively better in the comparison experiments, nine convergence curves obtained during the experiments are given below, as shown in Figure 13. The convergence curves of the COWOA have a convergence advantage over the other eight algorithms, except for the optimization F26, which finally achieves a relatively optimal convergence accuracy. In addition, the convergence curves of the eight compared algorithms are relatively smooth throughout the convergence process. In contrast, the convergence curves of the COWOA have different numbers of inflection points, and there is a very obvious drop in the curve after the inflection point, which indicates that the COWOA not only jumps out of the local optimum but also has a faster convergence speed. In summary, we can conclude that the convergence ability of the COWOA proposed in this paper is relatively more excellent compared to the eight excellent variants of other algorithms. Therefore, this experiment proves that the COWOA is an excellent swarm intelligence algorithm.

### Feature selection experiments

#### Experimental setup

To confirm the performance of the bCOWOA-KELM model in the direction of feature selection, this section conducts feature selection experiments based on six public datasets in the UCI and a medical dataset (HD) collected at the moment, respectively. To enhance the persuasiveness of the experimental results, this paper also uses nine binary swarm intelligence optimization algorithms in the feature selection experiments for comparison experiments with the bCOWOA, including bWOA, bGWO, bHHO, bMFO, bSCA, bSMA, bSSA, bCSO, and BBA. The experimental parameters of the corresponding algorithms are given in Table 13.

**TABLE 13:** Key parameters of the algorithms.

Algorithms	bCOWOA	bWOA	bGWO	bHHO	bMFO
**Values**	*a* = [0,2]	*a* = [0,2]	*a* = [0,2]	*E1* = [0,2]	*a* = 2
	*F* = 4			*E0* = [-1,1]	*b* = 1
	*Q* = 3				

**Algorithms**	**bSCA**	**bSMA**	**bSSA**	**bSCO**	**BBA**

**Values**	*r1* = [0,2]	*a* = [0,5]	∼	∼	*A* = 0.5
		*b* = [0,1]			*r* = 0.5

In the experimental section of the public datasets, we set up comparison experiments based on six public datasets from the UCI repository, which are used to validate the performance of the bCOWOA-KELM model on the feature selection problem. Since the medical data problem addressed in this paper is binary in nature, the public datasets selected for this section are all of the binary type. Details of the parameters of these six public datasets are presented in Table 14.

**TABLE 14:** Description of public datasets.

Datasets	Samples	Features	Classes
Breast	569	31	2
Ionosphere	351	35	2
HeartEW	270	14	2
Congress	435	17	2
Breastcancer	699	10	2
Heart	270	14	2

In the experimental section of the HD dataset, HD sessions were recorded from April 1, 2020, to April 18, 2020, in an outpatient HD unit at First Affiliated Hospital of Wenzhou Medical University. The inclusion criteria are as follows: (1) age ≥ 18 years; (2) maintenance HD ≥ 3 months; (3) the frequency of the treatment was three times per week, and the duration of the treatment was 4 hours. The exclusion criteria are as follows: (1) HD sessions with missing data; (2) HD therapy without using of heparin or low molecular heparin. A total of 156 patients with 1239 HD sessions were included. The dialysate temperature was 37^°^C, sodium concentration was 140 mmol/L, and calcium concentration was 1.5 mmol/L at the beginning of the sessions. Patients were detected blood routine once a month. The fast serological specimens were collected from a peripheral vein in a sitting position before dialysis on April 1st or April 2nd (mid-week therapy). BP was measured five times in each treatment session: At 0-h, 1 h, 2 h, 3 h after drawing blood and at reinfusion. Extra measurements of BP were carried out when patients suffered discomforts. IDH was defined as a drop of SBP ≥ 20 mmHg or a drop of (mean arterial pressure) MAP ≥ 10 mmHg from pre-dialysis to nadir intradialytic levels plus ≥ 2 repetitive measures (K/Doqi Workgroup, 2005). The description of this dataset’s details is given below, as shown in Table 15.

**TABLE 15:** Description of attributes screened by the classifiers.

No.	Feature	Detailed description
F1	Dialysis vintage (months)	Non-IDH group (median, IQR) = 68, 74 IDH group (median, IQR) = 100, 124
F2	Diabetes	No = 0; Yes = 1
F3	Ultrafiltration volume (kg)	Non-IDH group (median, IQR) = 2.0, 1 IDH group (median, IQR) = 2.2, 1
F4	Age (years old)	Non-IDH group (median, IQR) = 66, 18 IDH group (median, IQR) = 66, 13
F5	Dry weight (kg)	Non-IDH group (median, IQR) = 56.1, 14.7 IDH group (median, IQR) = 54.0, 12.9
F6	Pre-dialysis weight (Kg)	Non-IDH group (median, IQR) = 58.1, 14.5 IDH group (median, IQR) = 56.2, 13.9
F7	Interdialytic weight gain (kg)	Non-IDH group (median, IQR) = 1.9, 1 IDH group (median, IQR) = 2.1, 1
F8	Percentage of interdialytic weight gain (%)	Non-IDH group (median, IQR) = 3.46, 2.23 IDH group (median, IQR) = 3.87, 1.78
F9	Systolic blood pressure (mmHg)	Non-IDH group (median, IQR) = 133, 29 IDH group (median, IQR) = 150, 30
F10	Diastolic blood pressure (mmHg)	Non-IDH group (median, IQR) = 74, 15 IDH group (median, IQR) = 79, 17
F11	Mean arterial pressure (mmHg)	Non-IDH group (median, IQR) = 93, 18 IDH group (median, IQR) = 103, 19
F12	Heart rates (bpm)	Non-IDH group (median, IQR) = 76, 16 IDH group (median, IQR) = 77, 15
F13	Gender	Male = 1; female = 0
F14	White blood cell (10^9^/L)	Non-IDH group (median, IQR) = 5.39, 1.83 IDH group (median, IQR) = 5.25, 2.37
F15	Neutrophil (%)	Non-IDH group (median, IQR) = 65.0, 8.9 IDH group (median, IQR) = 64.4, 13.2
F16	Eosinophil (%)	Non-IDH group (median, IQR) = 3.3, 3.5 IDH group (median, IQR) = 3.1, 3.5
F17	Basophil (%)	Non-IDH group (median, IQR) = 0.1, 0.2 IDH group (median, IQR) = 0.2, 0.3
F18	Monocyte (%)	Non-IDH group (median, IQR) = 7.3, 3.2 IDH group (median, IQR) = 8.0, 3.5
F19	Lymphocyte (%)	Non-IDH group (median, IQR) = 22.0, 9.7 IDH group (median, IQR) = 22.3, 10.0
F20	Neutrophil to lymphocyte ratio	Non-IDH group (median, IQR) = 3.03, 1.70 IDH group (median, IQR) = 2.90, 1.92
F21	Monocyte to lymphocyte ratio	Non-IDH group (median, IQR) = 0.33, 0.19 IDH group (median, IQR) = 0.37, 0.22
F22	Platelet to lymphocyte ratio	Non-IDH group (median, IQR) = 145.5, 91.3 IDH group (median, IQR) = 145.5, 82.9
F23	Neutrophil to monocyte ratio	Non-IDH group (median, IQR) = 8.76, 4.48 IDH group (median, IQR) = 8.76, 5.06
F24	Red blood cell (10^12^/L)	Non-IDH group (median, IQR) = 3.64, 0.50 IDH group (median, IQR) = 3.57, 0.61
F25	Hemoglobin (g/L)	Non-IDH group (median, IQR) = 114, 13 IDH group (median, IQR) = 114, 16
F26	Hematocrit	Non-IDH group (median, IQR) = 0.34, 0.04 IDH group (median, IQR) = 0.35, 0.06
F27	Mean corpuscular volume (fL)	Non-IDH group (median, IQR) = 95.6, 5.3 IDH group (median, IQR) = 96.1, 6.1
F28	Mean corpuscular hemoglobin (pg)	Non-IDH group (median, IQR) = 31.3, 2.1 IDH group (median, IQR) = 31.0, 2.1
F29	Mean corpuscular hemoglobin concentration (g/L)	Non-IDH group (median, IQR) = 327, 10 IDH group (Median, IQR) = 324, 12
F30	Red cell volume distribution width (%)	Non-IDH group (median, IQR) = 13.8, 1.1 IDH group (median, IQR) = 14.3, 1.2
F31	SD of red cell volume distribution (fL)	Non-IDH group (median, IQR) = 48.1, 4.3 IDH group (Median, IQR) = 48.8, 6.2
F32	Platelet (10^9^/L)	Non-IDH group (median, IQR) = 177, 86 IDH group (median, IQR) = 187, 80
F33	Thrombocytocrit	Non-IDH group (median, IQR) = 0.19, 0.08 IDH group (median, IQR) = 0.19, 0.07
F34	Mean platelet volume (fL)	Non-IDH group (median, IQR) = 10.4, 1.2 IDH group (median, IQR) = 10.4, 1.1
F35	SD of platelet distribution (fL)	Non-IDH group (median, IQR) = 11.9, 2.4 IDH group (median, IQR) = 12.2, 2.5
F36	Platelet large cell ratio (%)	Non-IDH group (median, IQR) = 27.8, 9.8 IDH group (median, IQR) = 27.7, 9.0

IDH denotes intradialytic hypotension and IQR denotes interquartile range.

In addition, this paper analyses and compares the average value (Avg) and standard deviation (Std) of the experimental results and evaluates the comprehensive performance of each binary classification model by ranking them, thus demonstrating more intuitively that the bCOWOA-KELM model has relatively better feature selection performance.

Finally, to ensure the fairness of the experimental process of feature selection, we set the overall size of each algorithm population to 20 and the number of iterations to 100 times uniformly; to ensure the consistency of the experimental environment and to avoid the influence of environmental factors on the experimental results, the running environment of all experiments in this section is consistent with the experimental part of the basic function.

#### Performance evaluation metrics

In this subsection, we present some of the analytical evaluation methods used to analyze the results of the feature selection experiments (Hu et al., 2022a,b; Liu et al., 2022a,c; Shan et al., 2022; Shi et al., 2022; Xia et al., 2022a; Yang et al., 2022b; Ye et al., 2022). The aim is to provide a valid theoretical basis for demonstrating that the bCOWOA-KELM model performs better in feature selection than other comparative models. In the following, each of the mentioned evaluation methods is described.

In experiments, we usually classify the truth of the data as true (T) and false (F). We would then predict and classify them by machine learning, and the resulting positive predictions are defined as Positive (P) and the negative results are defined as Native (N). Thus, throughout the feature classification experiments, we typically derive four performance evaluation metrics that are used to assess the performance of the binary classifier model, as shown in Table 16.

**TABLE 16:** Description of classification details.

Class	Positive (P)	Native (N)
True (T)	TP	TN
False (F)	FP	FN

The following is a detailed description of Table 16.

(1) TP (True Positive): indicates a positive class prediction, where the classifier predicts the same data sample situation as the true one.
(2) FP (False Positive): The classifier misrepresents the negative class prediction as a positive class prediction, where the classifier discriminates the data sample situation as the opposite of the true situation and misidentifies the negative result as a positive result.
(3) TN (True Negative): indicates a negative class prediction and the classifier correctly identifies the negative class prediction.
(4) FN (False Negative): Positive class prediction is treated as a negative class prediction, resulting in the classifier missing the positive class prediction.

In addition, in order to better facilitate the evaluation of the feature extraction capability and classification capability of the bCOWOA-KELM model and to enhance the persuasiveness of the model proposed in this paper, this paper uses four categories of evaluation criteria commonly used in the fields of machine learning and information retrieval, and there are Accuracy, Specificity, Precision, and F-measure. The following section describes the details of the 4 evaluation criteria for the classifier experiments are described as follows:

(1) Accuracy indicates the number of samples successfully classified by the classifier as a proportion of all samples. In general, a larger accuracy rate indicates better performance of the classifier.

(39) $$A\,c\,c\,u\,r\,a\,c\,y=\frac{T\,P+T\,N}{T\,P+T\,N+F\,P+F\,N}$$

(2) Specificity indicates the proportion of all negative cases that are successfully classified, and measures the classifier’s ability to identify negative cases.

(40) $$S\,p\,e\,c\,i\,f\,i\,c\,i\,t\,y=\frac{T\,N}{T\,N+F\,P}$$

(3) Precision indicates the proportion of true positive prediction instances among all instances discriminated by the classifier as positive prediction outcomes.

(41) $$P\,r\,e\,c\,i\,s\,i\,o\,n=\frac{T\,P}{T\,P+F\,P}$$

(4) F-measure (F) is a special comprehensive evaluation criterion among the several types of evaluation criteria mentioned in this section, and is used to evaluate the overall performance of the binary model in the same way as error rate and accuracy. Its evaluation value is the weighted average of precision (*P*) and recall (*R*).

(42) $$R=\frac{T\,P}{T\,P+F\,N}$$

(43) $$F=\frac{\left(\alpha^{2}+1\right)\,P\cdot R}{\alpha^{2}\,\left(P+R\right)}$$

where α generally takes a value of 1; *P* denotes Precision as mentioned above; and *R* denotes Recall, which is an assessment criterion covering the range ability and describes the underreporting of positive class predictions and the degree of recall of positive class results.

#### Public dataset experiment

In this section, we set up comparative experiments based on six public datasets from the UCI repository to verify that the comprehensive performance of the bCOWOA-KELM model is relatively optimal in this experiment and also demonstrate that the bCOWOA-KELM model is more adaptive. For the experimental analysis, three evaluation methods were selected. The results were analyzed and validated by the Avg and Std design in each evaluation method and the average ranking of the algorithms involved in the comparison experiment on the six public datasets. The experimental analysis is presented below.

The experimental results of the classification accuracy of the bCOWOA-KELM model compared with other feature selection methods are given in Supplementary Table 5. The table shows the average classification of the bCOWOA-KELM model on the Breast, Ionosphere, HeartEW, Congress, Breastcancer, and heart datasets. The accuracy was always the largest, and their average classification accuracies were all above 94.81%, indicating that the bCOWOA-KELM model has relatively optimal classification ability. To further enhance the convincing power, the average ranking of each algorithm on the six public datasets was counted in this experiment, as shown in Table 17. It can be seen that bCOWOA ranks first in terms of average classification accuracy, which indicates that bCOWOA has outstanding adaptability to different datasets; BBA has the worst average ranking, which indicates that BBA has the relatively worst adaptability.

**TABLE 17:** Average classification accuracy ranking.

Algorithms	bCOWOA	bWOA	bGWO	bHHO	bMFO	bSCA	bSMA	bSSA	bCSO	BBA
Rank-Avg	**1**	5.33	9.17	5.67	3.83	4.5	4.83	6.33	4.5	9.83
Rank	**1**	5	8	6	2	3	4	7	3	9

The results of this experiment describing the precision are given in Supplementary Table 6. As seen from the table, the average of bCOWOA on the six public datasets is always relatively the largest and its average precision is above 93.36%, which indicates that the bCOWOA-KELM model has the relatively best rate of correct classification among the compared methods. In addition, we also present the average ranking of each algorithm on the six datasets, as shown in Table 18. In particular, the bCOWOA-KELM model ranked first in terms of accuracy, bSMA ranked second, and bSCA ranked last.

**TABLE 18:** Average classification precision ranking.

Algorithms	bCOWOA	bWOA	bGWO	bHHO	bMFO	bSCA	bSMA	bSSA	bCSO	BBA
Rank-Avg	**1**	6	6.83	5.17	5.83	8.83	4.67	5.17	5.17	6.33
Rank	**1**	5	7	3	4	8	2	3	3	6

Supplementary Table 7 shows the mean F-measure and variance of the bCOWOA-KELM model and the nine comparison models participating in the experiment on six public datasets. The F-measure is known to be a comprehensive criterion for assessing the classification performance of an algorithm, and it provides a more comprehensive assessment of the classification capability of bCOWOA. As can be seen from the table, the average F-measure values of bCOWOA are consistently the largest and their average accuracy is above 95.39%. As shown in Table 19, we also give the average ranking of each classification method on the six datasets. It can be seen that the bCOWOA-KELM model ranks first on average in terms of F-measure.

**TABLE 19:** Average ranking of F-measure.

Algorithms	bCOWOA	bWOA	bGWO	bHHO	bMFO	bSCA	bSMA	bSSA	bCSO	BBA
Rank-Avg	**1**	5.5	9.17	4.83	4.83	4.33	4.33	6.17	5	9.83
Rank	**1**	5	7	3	3	2	2	6	4	8

#### Hemodialysis dataset experiment

In this section, in order to verify whether the bCOWOA-KELM model is effective, we collected information such as the clinical features, dialysis parameters and indexes of blood routine test from the dataset. We conducted prediction IDH comparison experiments on the bCOWOA-KELM model proposed in this paper. To analytically validate the performance of the proposed bCOWOA-KELM model, four different classifier evaluation metrics were used to assess the comprehensive performance of the model as far as possible, including Accuracy, Specificity, Precision and F-measure. To demonstrate that the bCOWOA-KELM model is superior, we compare bCOWOA-KELM with combinations of bCOWOA and four other classical classifiers, including bCOWOA-FKNN, bCOWOA -KNN, bCOWOA-MLP, and bCOWOA -SVM. To further compare the performance differences between the categorical prediction model based on swarm intelligence optimization algorithm and classical machine learning algorithms such as RandomF, AdaBoost, and CART, we set up a comparison experiment between the bCOWOA-KELM model and these methods. Furthermore, to demonstrate that the predictive performance of the combination of bCOWOA-KLEM in the swarm intelligence optimization algorithms is also relatively better, we selected nine well-known swarm intelligence algorithms to set up a comparison experiment of with bCOWOA in this section, such as bGWO, bHHO, bSMA and so on. Finally, in order to demonstrate that the bCOWOA-KELM model has practical application value in the prediction of IDH and to reduce the effect of random factors on the experiment, we set up a 10-fold cross-validation (CV) analysis experiment on it.

**FIGURE 14:**
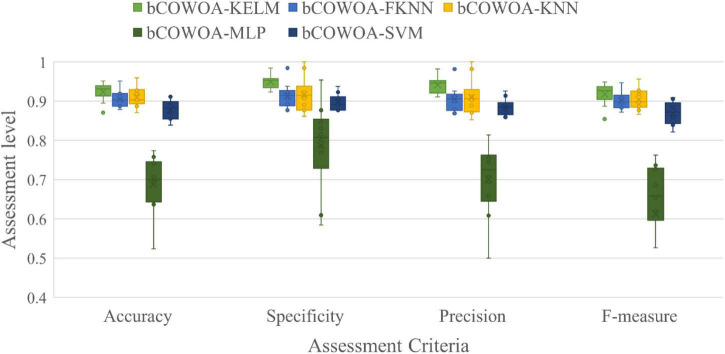
Comparison results of the five bonding methods.

In general, the same binary algorithm combined with different classifier methods often produces different classification results. The comparison experiment of the bCOWOA-KELM model with four other classifier combinations, including bCOWOA-FKNN, bCOWOA -KNN, bCOWOA-MLP, and bCOWOA -SVM, the analysis of the results for the four evaluation methods is given in a box plot in Figure 14. The graph shows that the values of the four evaluation criteria for the bCOWOA-KELM model are relatively more concentrated and have the relatively highest mean values. This indicates that the combined approach has the relatively best classification prediction ability and the most stable classification performance. bCOWOA-MLP model is the worst in the four aspects. The bCOWOA-FKNN model and the bCOWOA-KELM model perform similarly. However, looking closely at the box diagram, it is not difficult to find that the bCOWOA-KELM classification combination model has better means on all evaluation criteria, and the evaluation data generated by the experiment is relatively more concentrated. This shows that the bCOWOA-KELM is not only better at the end result but also proves that it has stronger stability. Therefore, we can conclude that the bCOWOA-KELM model is the best classification prediction model among the five combined approaches.

**FIGURE 15:**
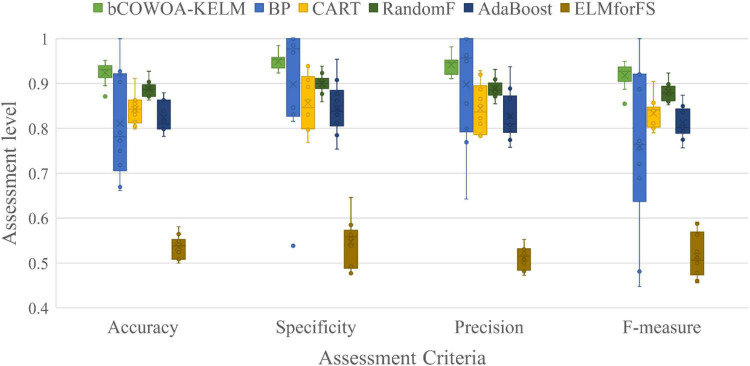
Comparison results of the bCOWOA-KELM with five classifiers.

In order to examine the effect of the swarm intelligence optimization algorithm on the classification performance of the classifier in terms of feature selection experiments, we compared the bCOWOA-KELM model with five machine learning methods that did not incorporate the bCOWOA, and the comparison results are shown in Figure 15. Compared with BP, the proposed bCOWOA-KELM model has better stability than BP, although it is less optimal. Compared with the other four classification methods, the proposed bCOWOA-KELM model is better in terms of Accuracy and specificity and has a more stable performance. In summary, the comprehensive performance of the bCOWOA-KELM model proposed in this paper is better than the original classification method without the swarm intelligence algorithm.

To demonstrate the practical relevance of the prediction model and to further verify that the performance of the bCOWOA-KELM model is relatively optimal on the IDH dataset, the proposed bCOWOA was compared with nine binary algorithms based on the KELM classification technique, including bWOA, bGWO, bHHO, bMFO, bSCA, bSMA, bSSA, Bcso, and BBA. Then, all combinations of the above binary algorithms and KELM were analyzed and evaluated in terms of six aspects: Accuracy, Specificity, Precision, F-measure, Error, and time spent.

**FIGURE 16:**
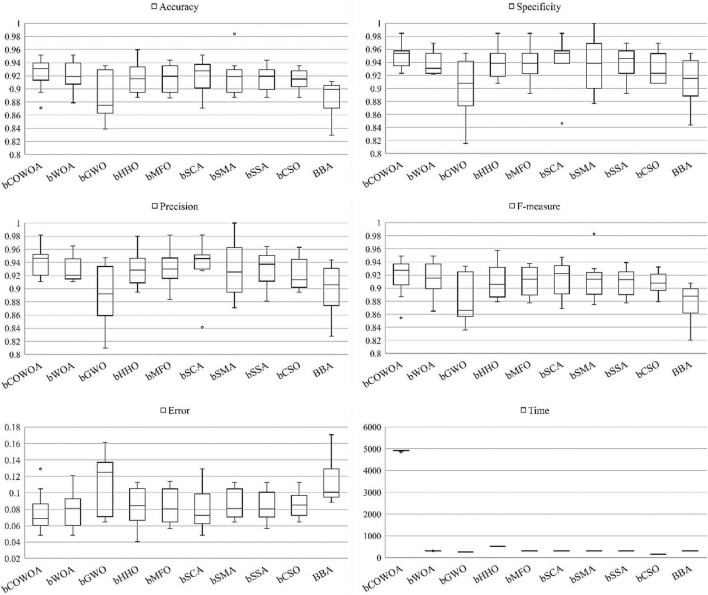
Comparison results of bCOWOA with other binary algorithms.

The statistical results of this experiment on the HD dataset are given in Figure 16. According to the results, the combination of bCOWOA and KELM on the first five evaluation criteria have a relatively concentrated box plot distribution compared to the combination of the other nine algorithms, indicating that the bCOWOA-KELM model has very considerable stability compared to the other algorithms. Meanwhile, bCOWOA also has the highest average value, indicating that bCOWOA has better classification capability than all the algorithms compared. However, the bCOWOA’s average value is the highest in terms of time spent, which is one of the drawbacks that should be of concern for the future of the algorithm. In conclusion, this part of the experiment demonstrates that the bCOWOA-KELM model has the relatively best classification ability for the HD dataset.

Table 20 analyses the results of the bCOWOA-KELM model after completing 10 feature selection experiments. In rows 2–11 of Table 20, the Fold column indicates the number of experiments, the second column indicates the number of features selected in each feature selection experiment, the remaining four columns indicate the Accuracy, Specificity, Precision and F-measure obtained, and the last two rows of Table 20 give the average value and variance corresponding to the four evaluation criteria, respectively. As can be seen from the table, the average classification accuracy value of the bCOWOA-KELM is 0.9241, the average specificity value is 0.9492, the average accuracy value is 0.9415 and the average F-measure is 0.9180. According to Table 21, it is easy to find that bCOWOA obtains the better scores on the four metrics In addition, bCOWOA improves 0.32% in accuracy, 0.62% in specificity, 0.54% in Precision, and 0.29% in F-measure over the second-ranked bSCA. bCOWOA improved 3.63% in accuracy, 4.62% in specificity, 4.94% in Precision, and 3.70% in F-measure over the worst-ranked bGWO.

**TABLE 20:** Results of the bCOWOA-KELM model.

Fold	Number of features selected	Accuracy	Specificity	Precision	F-measure
#1	17	0.9516	0.9846	0.9818	0.9474
#2	16	0.9516	0.9539	0.9492	0.9492
#3	16	0.9194	0.9692	0.9623	0.9107
#4	24	0.9355	0.9231	0.9180	0.9333
#5	20	0.9194	0.9539	0.9455	0.9123
#6	24	0.8952	0.9231	0.9107	0.8870
#7	16	0.9350	0.9531	0.9474	0.9310
#8	20	0.9274	0.9385	0.9310	0.9231
#9	17	0.9355	0.9539	0.9474	0.9310
#10	17	0.8710	0.9385	0.9216	0.8546
AVG	∼	**0.9241**	**0.9492**	**0.9415**	**0.9180**
STD.	∼	0.0250	0.0192	0.0216	0.0289

**TABLE 21:** The average of bCOWOA and well-known binary algorithms.

	Accuracy		Specificity		Precision		F-measure	
	**AVG**		**AVG**		**AVG**		**AVG**	
**bCOWOA**	**0.9241**	**92.41%**	**0.9492**	**94.92%**	**0.9415**	**94.15%**	**0.9180**	**91.80%**
bWOA	0.9201	92.01%	0.9368	93.68%	0.9286	92.86%	0.9145	91.45%
bGWO	0.8878	88.78%	0.9030	90.30%	0.8921	89.21%	0.8810	88.10%
bHHO	0.9169	91.69%	0.9383	93.83%	0.9302	93.02%	0.9105	91.05%
bMFO	0.9168	91.68%	0.9399	93.99%	0.9316	93.16%	0.9107	91.07%
bSCA	0.9209	92.09%	0.9430	94.30%	0.9361	93.61%	0.9152	91.52%
bSMA	0.9193	91.93%	0.9338	93.38%	0.9272	92.72%	0.9142	91.42%
bSSA	0.9153	91.53%	0.9414	94.14%	0.9327	93.27%	0.9088	90.88%
bCSO	0.9136	91.36%	0.9307	93.07%	0.9221	92.21%	0.9079	90.79%
BBA	0.8878	88.78%	0.9105	91.05%	0.8988	89.88%	0.8799	87.99%

**FIGURE 17:**
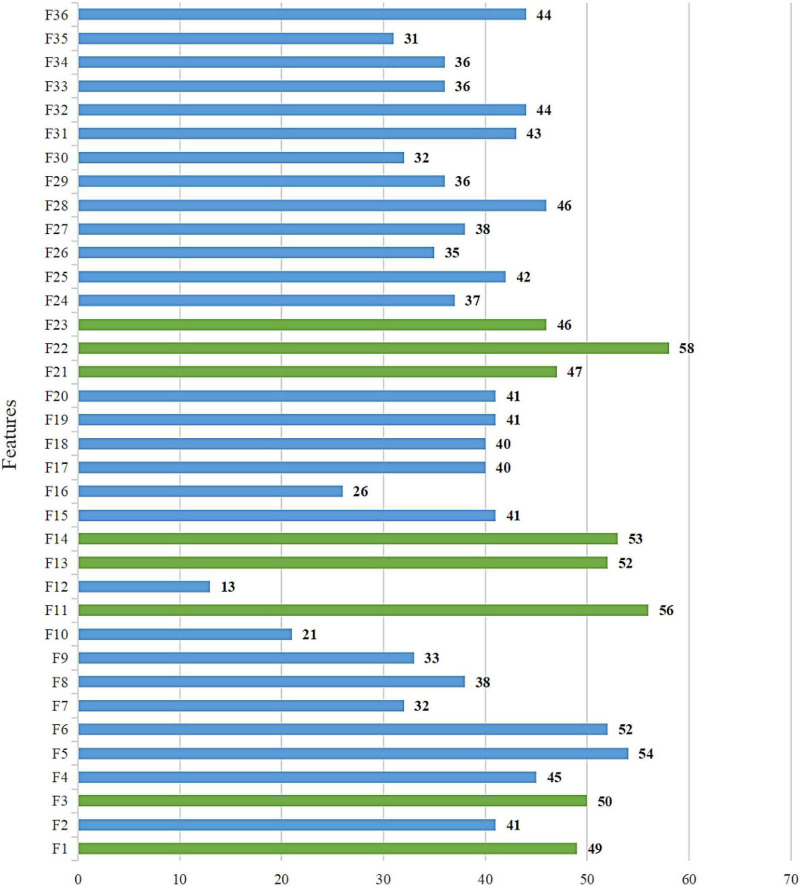
Results of 10 times 10-fold cross-validation analysis.

To further verify the predictive performance of the bCOWOA-KELM model, we conducted a 10 times 10-fold crossover experiments based on the HD dataset. To facilitate the selection of features and the analysis of experimental results, we present 36 features and their selection through a histogram, as shown in Figure 17. What’s more, the features selected by bCOWOA-KELM to predict IDH are illustrated in Table 22.

**TABLE 22:** Clinical features selected by in IDH and Non-IDH groups.

Selected features index	Non-IDH group (*n* = 649)	IDH group (*n* = 590)	*p*-value
Platelet to lymphocyte ratio (median, IQR)	145.5, 91.3	145.5, 82.9	0.930
Mean arterial pressure (median, IQR, mmHg)	93, 18	103, 19	<0.001
White blood cell (median, IQR, 10^9^/L)	5.39, 1.83	5.25, 2.37	0.409
Gender (male %)	59%	55%	0.197
Ultrafiltration volume (median, IQR, kg)	2.0, 1	2.2, 1	0.001
Dialysis vintage (median, IQR, months)	68, 74	100, 124	<0.001
Monocyte to lymphocyte ratio (median, IQR)	0.33, 0.19	0.37, 0.22	0.144
Neutrophil to monocyte ratio (median, IQR)	8.76, 4.48	8.76, 5.06	0.006

IDH denotes intradialytic hypotension and IQR denotes an interquartile range.

In Figure 17, the vertical axis represents the code name of the feature that appeared in this experiment and the abscissa axis represents the number of times each feature was selected in 100 experiments. The characteristics of the green color in the figure are the key features selected in this experiment, including F22, F11, F14, F13, F3, F1, F21, and F23. They, respectively, represent platelet to lymphocyte ratio (PLR), MAP, white blood cells (WBCs), gender, ultrafiltration volume, dialysis vintage, monocyte to lymphocyte ratio (MLR) and neutrophil to monocyte ratio (NMR). Finally, we concluded that the eight key features selected by the bCOWOA-KELM model in this experiment are in line with clinical practice. Therefore, we have once again demonstrated the effectiveness of the bCOWOA-KELM model in combination with the clinical experience of IDH.

## Discussion

### Comparison with previous studies and summary

Although the definition of IDH was different in various studies, even in recent studies, IDH was defined as an SBP below to 90 mmHg, to build an early warning system, IDH was defined according to guidelines of the National Kidney Foundation Kidney Disease Quality Outcomes Initiative (K/DOQI) (K/Doqi Workgroup, 2005). To predict IDH episodes, previous studies analyzed pre-dialysis BP, demographic characteristics and ultrafiltration parameters. Liu et al. (2022e) study applied the least absolute shrinkage and selection operator (LASSO) to select features from dialysis parameters and patients’ characteristics. The sensitivity and specificity were below 90%. Some of them also included serum biomarkers in the analysis to improve accuracy. Assayag et al. (2020) analyzed B-natriuretic peptide using a logistic mixed model to predict the 30-day risk of IDH. The accuracy was still not satisfactory. Our previous study got good accuracy from an artificial neural network (ANN) model utilizing chronic kidney disease-mineral and bone disorders (CKD-MBD) biomarkers. The biomarkers mentioned above are associated with volume or vasoactivity, which are determinants of BP. Compared with these biomarkers, blood routine test is generally carried out in basic hospitals and are inexpensive, though does not have a direct correlation with BP. The prediction models are divided into logistic regression models and ANN models. As we know, the events are not linearly dependent on features in clinical practice. Various ANN models are apt to deal with non-linear relationships.

To predict IDH, our team built different models to screen various factors. Yang et al. (2022a) proposed an IDH prediction model (BSWEGWO-KELM) based on improved GWO and KELM. And this method successfully screened four key features that affect the incidence of IDH, including dialysis vintage, MAP, alkaline phosphatase (ALP), and intact parathyroid hormone (iPTH). Hu et al. (2022c) proposed a promising model (MQGWO-FKNN) using the Fuzzy K-Nearest Neighbor (FKNN) based on the mutation quantum GWO, and the method enabled the prediction of serum albumin increases and decreases to assist in the diagnosis of IDH. Also, the key features selected by the MQGWO-FKNN model were analyzed with physiological significance, including age, dialysis vintage, diabetes, and baseline albumin. It is worth noting that although the methods mentioned above compensate for the performance shortcomings of a single technique by enabling the combination of swarm intelligence optimization algorithms with classical classification techniques and achieve prediction of the influencing factors of IDH, it is not difficult to find crossover and complementarity between the classification results of the above two methods, which shows that the factors that affect IDH are not a single subset of features and also illustrates the real and non-linear relationship between clinical features and IDH.

Therefore, in order to further explore those clinical features that are more relevant to IDH morbidity, this paper proposes another novel classification approach (bCOWOA-KELM) to predict IDH. And unlike the former, the dataset collected this time has been adjusted in terms of features and number of features. Finally, the experimental results show that the proposed method successfully predicts eight key characteristics affecting IDH. Compared with the former two, the features predicted by the bCOWOA-KELM model are more abundant, which once again supplements the experimental basis to predict IDH and expands the direction of related research in the future. At the same time, this paper once again verifies that the clinical characteristics and IDH incidence are not linear, which is also one of the important practical medical significances of this paper. In addition, the features screed by bCOWOA-KELM are contain indices of blood routine test. Compared with the former study, blood routine test is easier to use and more cost-efficient biomarkers than CKD-MBD.

### Physiological significance of the selected features

In this study, COWOA is first built, which is a verified excellent swarm intelligence algorithm. This class of methods can be applied many fields, such as dynamic module detection (Ma et al., 2020; Li et al., 2021), power flow optimization (Cao X. et al., 2022), information retrieval services (Wu et al., 2020a,b, 2021c), human activity recognition (Qiu et al., 2022), location-based services (Wu et al., 2020c,2021b), disease identification and diagnosis (Su et al., 2019; Tian et al., 2020), pharmacoinformatic data mining (Zhu et al., 2012; Yin et al., 2020), autism spectrum disorder classification (Hu et al., 2022d), endoscope imaging (Zhang Z. et al., 2022), and image-to-image translation (Zhang X. et al., 2022). Then, bCOWOA-KELM based on COWOA was also established. The critical features selected by bCOWOA-KELM were PLR, MAP, WBC, gender, ultrafiltration volume, dialysis vintage, MLR, and NMR.

Female gender is a risk factor of IDH assessed by logistic regression in previous studies (Sands et al., 2014; Halle et al., 2020). Females always have smaller body sizes than males; thus, even if the IDWG is the same in males and females, the percentage of IDWG and the ultrafiltration rates are higher in females than males. To get a good clinical outcome and avoid IDH, the suggested percentage of IDWG is below 4% (Wong et al., 2017), and the ultrafiltration rates is below 10 mL/h/kg (Flythe et al., 2011). The comorbidities and complications of CKD damage peripheral vascular resistance with dialysis vintage. First, baroreceptor variability is impaired by uremia toxins. Second, CKD-MBD and diabetes induce vascular calcification and aggravate atherosclerosis and arterial stiffness. Third, ß2 microglobulin amyloid deposits in cardiac myocytes and blood vessel wall (Takayama et al., 2001), impairs cardiac output and peripheral resistance. Cardiac output and peripheral resistance compensate for BP when blood volume is reduced during dialysis.

High MAP is equal to high BP. High BP causes cardiovascular injury. High BP is associated with coronary artery disease (Yeo et al., 2020), cardiac systolic dysfunction, and left ventricular hypertrophy (Chao et al., 2015). There are cause-and-effect relationships between these factors and IDH. High BP also induces endothelial dysfunction and arterial stiffness. In a clinical trial, both of them are independent risk factors of IDH (Dubin et al., 2011). The ultrafiltration volume is associated with IDH, especially after the first 90 min session (Keane et al., 2021).

PLR, WBC, MLR, and NMR represent inflammation and predict prognosis of cancer (Kumarasamy et al., 2021), infective (Palladino, 2021) and inflammatory diseases (Gasparyan et al., 2019). There are also relationships between these hematological indices and cardiac abnormalities. For example, increased MLR is an independent risk factor of death in patients with hypertension (Boos et al., 2021) or heart failure (Delcea et al., 2021). Our study demonstrated the hematological indices and IDH for the first time. Platelets, monocytes, and neutrophils are classified as pro-inflammatory cells, while lymphocytes are anti-inflammatory. These cells interact with each other. Since the parameters contain leukocyted, neutrophils, monocytes, lymphocytes, and platelets, we hypothesize the cytokines derived from these blood cells play roles in regulating BP. Yu et al. (2021) found the levels of serum tumor necrosis factor-α (TNF-α) and interleukin-1β (IL-1β) were higher in the IDH group. TNF-α and IL-1β are pro-inflammatory cytokines secreted by myeloid cells and able to activate platelet. These blood cells and cytokines regulate BP through cross-talk with renal renin-angiotensin system, sympathetic system, and oxidative stress (Zhang R. M. et al., 2021).

## Conclusion and future works

The main contributions of the present study are as follows: (1) a novel WOA-based swarm intelligence optimization algorithm was proposed, named COWOA, (2) a new IDH disease early warning model by combining the binary COWOA and the KELM was proposed, named bCOWOA-KELM, (3) the classification potential of KELM was successfully tapped based on the improved WOA and (4) the key features influencing the incidence of IDH are accurately identified using the bCOWOA-KELM model. PLR, WBC, MLR, and NMR are readily available, easy to use, and cost-efficient biomarkers, especially for those basic HD centers.

In COWOA, we successfully improved the search capability and the ability to escape local optima of the original WOA by introducing the OLM and the CMS into the WOA. To verify its performance, we set up four comparison experiments based on 30 benchmark test functions successively, including the comparison experiments between WOA and two mechanisms under different combinations, the comparison experiments between COWOA and seven excellent variants of WOA, the comparison experiments between COWOA and nine other original algorithms and the comparison experiments between COWOA and eight excellent variants of other algorithms. Based on the results of the above comparison experiments, the convergence ability of the COWOA is relatively the best compared to the other comparison algorithms. Therefore, The COWOA is an improved validated variant of WOA.

In bCOWOA-KELM, it is used for clinical prediction. First, to validate the performance of the bCOWOA-KELM model and its effectiveness, we set up two types of classification prediction experiments, including comparison experiments based on six public datasets and comparison experiments based on the HD dataset, and validated the classification results with Accuracy, Specificity, Precision and F- measure as the evaluation criteria to validate the comprehensive performance of the bCOWOA-KELM model. Second, we selected eight features based on the feature selection results of the HD dataset. Finally, the clinical significance of the eight characteristics is discussed in detail. The value of the bCOWOA-KELM model in disease prediction was further confirmed by all the features selected by it were comprehensible for nephrologist.

However, there are still a few limitations in our study, mainly including the COWOA itself and the HD dataset. For the COWOA, since the method proposed in this paper is based on the improved WOA, the introduction of CMS and OLM optimization methods greatly improves the performance of WOA, but it also affects the complexity of the proposed model, which results in it needing to spend more time cost to exert its stronger performance. For the HD dataset, its shortcomings are mainly manifested in three aspects, including: (1) the sample size was small, (2) the blood routine was only detected once to predict a half-month risk of IDH, and (3) IDH was divided into two groups without an order of severity or time-dependent. Therefore, we will further improve this design from the above aspects in the future.

In the future, we will solve the complexity of the COWOA, for example, (1) starting from the complex, under the premise of ensuring its performance, we continue to improve the COWOA, and (2) distributed computing, high-performance computing, and other advanced technologies should be applied in the process of disease prediction. Moreover, the IDH prediction model will be more available. Furthermore, an intelligent HD management system will be built based on an improved algorithm. In addition, we will also explore other application areas of COWOA, such as image segmentation (Liu L. et al., 2021; Zhao D. et al., 2021), engineering optimization (Qi et al., 2022; Qiao et al., 2022), resource allocation (Deng et al., 2022a).

## Data availability statement

The original contributions presented in the study are included in the article/Supplementary material, further inquiries can be directed to the corresponding author/s.

## Author contributions

YPL, GL, YL, YB, AI, and CW: writing—original draft, writing—review and editing, software, visualization, and investigation. DZ, HC, and XC: conceptualization, methodology, formal analysis, investigation, writing—review and editing, funding acquisition, and supervision. All authors contributed to the article and approved the submitted version.
