# Supplementary material for: Intradialytic hypotension prediction using covariance matrix-driven whale optimizer with orthogonal structure-assisted extreme learning machine
Source: Front Neuroinform. 2022 Oct 31;16:956423. doi: 10.3389/fninf.2022.956423 (PMC9659657; doi:10.3389/fninf.2022.956423)
Supplement: Supplementary file 1 [file Data_Sheet_1.pdf]

Appendix A

Table A.1 The comparison results between the COWOA optimization mechanisms

	F1		F2		F3	
	AVG	STD	AVG	STD	AVG	STD
COWOA	**1.00000E+02**	**1.27710E-08**	**2.00000E+02**	**1.18015E-14**	**3.00000E+02**	**2.79274E-14**
CMWOA	1.00000E+02	5.99808E-08	2.00000E+02	1.49278E-14	3.00000E+02	3.65655E-14
OWOA	4.02636E+07	2.36665E+07	1.78008E+07	2.39757E+07	1.01453E+04	4.39150E+03
WOA	2.77414E+07	1.07998E+07	3.93598E+06	5.62174E+06	3.04952E+04	2.13863E+04
	F4		F5		F6	
	AVG	STD	AVG	STD	AVG	STD
COWOA	4.00510E+02	8.91810E-01	**5.20000E+02**	8.47442E-05	6.26653E+02	3.64483E+00
CMWOA	**4.00207E+02**	**2.93063E-01**	5.20000E+02	**2.13543E-05**	6.34437E+02	3.59346E+00
OWOA	5.44870E+02	5.23839E+01	5.20828E+02	1.57909E-01	**6.26228E+02**	3.88876E+00
WOA	5.94236E+02	5.72555E+01	5.20377E+02	1.53305E-01	6.35182E+02	**3.21422E+00**
	F7		F8		F9	
	AVG	STD	AVG	STD	AVG	STD
COWOA	**7.00000E+02**	**5.97113E-14**	8.80028E+02	2.31083E+01	**1.06055E+03**	3.90938E+01
CMWOA	7.00034E+02	4.56648E-02	9.84365E+02	4.28827E+01	1.13189E+03	4.73962E+01
OWOA	7.01129E+02	7.21654E-02	**8.53602E+02**	**1.33904E+01**	1.06319E+03	**3.70172E+01**
WOA	7.01017E+02	5.69388E-02	9.94527E+02	3.26504E+01	1.13002E+03	6.55493E+01
	F10		F11		F12	
	AVG	STD	AVG	STD	AVG	STD
COWOA	**2.18862E+03**	**4.95438E+02**	**4.72226E+03**	7.13672E+02	**1.20031E+03**	**2.53095E-01**
CMWOA	4.31894E+03	8.22637E+02	5.25396E+03	**6.39244E+02**	1.20039E+03	2.67300E-01
OWOA	2.47069E+03	8.30755E+02	5.46770E+03	8.35122E+02	1.20172E+03	7.08060E-01
WOA	4.99933E+03	6.22260E+02	6.14939E+03	1.01904E+03	1.20149E+03	4.85780E-01
	F13		F14		F15	
	AVG	STD	AVG	STD	AVG	STD
COWOA	1.30056E+03	1.09956E-01	1.40029E+03	**4.50927E-02**	**1.52868E+03**	1.23540E+01
CMWOA	1.30051E+03	1.25204E-01	1.40030E+03	1.15631E-01	1.56796E+03	2.53347E+01
OWOA	1.30054E+03	1.24301E-01	**1.40028E+03**	5.57627E-02	1.53543E+03	**1.14222E+01**
WOA	**1.30050E+03**	**1.09294E-01**	1.40029E+03	1.17025E-01	1.57716E+03	3.54667E+01
	F16		F17		F18	
	AVG	STD	AVG	STD	AVG	STD
COWOA	**1.61208E+03**	6.62489E-01	**3.29000E+03**	**4.09307E+02**	2.03624E+03	**7.62688E+01**
CMWOA	1.61256E+03	5.43752E-01	3.45629E+03	4.14791E+02	**2.03089E+03**	9.55745E+01
OWOA	1.61223E+03	5.38685E-01	2.44985E+06	1.15679E+06	4.52897E+04	1.24911E+05
WOA	1.61247E+03	**5.25733E-01**	5.00396E+06	3.10647E+06	5.84876E+03	5.46564E+03
	F19		F20		F21	
	AVG	STD	AVG	STD	AVG	STD
COWOA	1.91453E+03	1.31696E+01	2.27619E+03	**1.08517E+02**	3.14790E+03	3.33444E+02
CMWOA	**1.91438E+03**	5.71095E+00	**2.26013E+03**	1.24316E+02	**3.10403E+03**	**2.57457E+02**
OWOA	1.91879E+03	**3.33220E+00**	6.87455E+03	2.88111E+03	8.75875E+05	5.25353E+05
WOA	1.95217E+03	4.59619E+01	2.80290E+04	2.15656E+04	1.32546E+06	1.15718E+06
	F22		F23		F24	
	AVG	STD	AVG	STD	AVG	STD

COWOA	2.81909E+03	2.08250E+02	**2.61401E+03**	**0.00000E+00**	2.60194E+03	7.41107E+00
CMWOA	2.96159E+03	2.79512E+02	2.61401E+03	0.00000E+00	**2.60012E+03**	**3.45374E-01**
OWOA	**2.75684E+03**	**1.61584E+02**	2.61840E+03	3.37790E+00	2.60377E+03	9.67453E+00
WOA	2.95325E+03	2.02617E+02	2.62890E+03	2.52772E+01	2.60506E+03	3.35629E+00
	F25		F26		F27	
	AVG	STD	AVG	STD	AVG	STD
COWOA	2.71563E+03	1.14667E+01	2.70383E+03	1.81646E+01	**3.60110E+03**	**2.71126E+02**
CMWOA	**2.71072E+03**	1.13018E+01	2.70375E+03	1.81787E+01	3.76069E+03	3.44245E+02
OWOA	2.71162E+03	**7.20987E+00**	**2.70058E+03**	**9.19990E-02**	3.61373E+03	3.12662E+02
WOA	2.71934E+03	1.72602E+01	2.71680E+03	5.77592E+01	3.70293E+03	3.72817E+02
	F28		F29		F30	
	AVG	STD	AVG	STD	AVG	STD
COWOA	4.38974E+03	6.51955E+02	3.23135E+03	1.81201E+02	3.92880E+03	3.06485E+02
CMWOA	4.65452E+03	5.20916E+02	3.19031E+03	1.55010E+02	**3.81026E+03**	**2.38599E+02**
OWOA	**3.35669E+03**	**1.08511E+02**	**3.12407E+03**	**1.95015E+01**	3.99341E+03	5.08458E+02
WOA	4.99367E+03	5.60435E+02	5.45626E+06	4.87365E+06	8.42411E+04	5.13852E+04

Table A.2 Comparison results of COWOA with the well-known WOA variant algorithms

	F1		F2		F3	
	AVG	STD	AVG	STD	AVG	STD
COWOA	**1.00000E+02**	**2.97535E-07**	**2.00000E+02**	**9.14138E-15**	**3.00000E+02**	**3.33796E-14**
ACWOA	1.20964E+08	5.78154E+07	7.80873E+09	4.09693E+09	5.02097E+04	8.03577E+03
IWOA	5.29741E+06	3.30220E+06	3.09265E+04	3.24942E+04	4.94845E+03	3.41244E+03
BMWOA	1.10559E+08	4.35634E+07	2.02695E+08	8.85870E+07	5.31083E+04	9.04509E+03
LWOA	3.76260E+06	1.60555E+06	5.75106E+05	1.56222E+05	9.47659E+02	2.91279E+02
EWOA	3.52647E+06	2.37385E+06	1.10770E+04	1.09944E+04	5.13246E+03	4.09478E+03
CWOA	7.77313E+07	4.78438E+07	2.92968E+09	3.37195E+09	6.58750E+04	3.77036E+04
OBWOA	5.17685E+07	2.05665E+07	5.66641E+07	7.34724E+07	2.79392E+04	1.15471E+04
	F4		F5		F6	
	AVG	STD	AVG	STD	AVG	STD
COWOA	**4.00792E+02**	**1.27426E+00**	**5.20000E+02**	**4.32748E-05**	6.26116E+02	4.16965E+00
ACWOA	1.21237E+03	2.63327E+02	5.20831E+02	1.37062E-01	6.32672E+02	2.97075E+00
IWOA	5.16776E+02	3.64355E+01	5.20102E+02	9.42488E-02	**6.22382E+02**	3.52636E+00
BMWOA	6.74263E+02	5.60502E+01	5.20974E+02	9.96511E-02	6.33196E+02	3.60141E+00
LWOA	5.11766E+02	3.50608E+01	5.20483E+02	9.26001E-02	6.29151E+02	**2.90421E+00**
EWOA	5.11844E+02	3.99403E+01	5.20127E+02	9.51922E-02	6.23267E+02	3.40677E+00
CWOA	8.07436E+02	1.93785E+02	5.20299E+02	1.24416E-01	6.35386E+02	3.14467E+00
OBWOA	6.37599E+02	5.60402E+01	5.20668E+02	1.40129E-01	6.32425E+02	3.23168E+00
	F7		F8		F9	
	AVG	STD	AVG	STD	AVG	STD
COWOA	**7.00000E+02**	**6.33333E-14**	8.81520E+02	2.09502E+01	1.08201E+03	4.22482E+01
ACWOA	7.39128E+02	1.99195E+01	9.89246E+02	1.98882E+01	1.12690E+03	**2.47984E+01**
IWOA	7.00188E+02	1.53469E-01	**8.33287E+02**	1.28948E+01	**1.06546E+03**	3.58511E+01
BMWOA	7.02958E+02	8.25264E-01	9.63918E+02	1.50278E+01	1.12201E+03	3.12562E+01
LWOA	7.00670E+02	1.00179E-01	8.73058E+02	1.60009E+01	1.12040E+03	5.54413E+01
EWOA	7.00057E+02	7.14849E-02	8.34728E+02	**1.11262E+01**	1.06659E+03	4.35636E+01
CWOA	7.12021E+02	1.56365E+01	1.01097E+03	4.69609E+01	1.14630E+03	6.15626E+01
OBWOA	7.01350E+02	1.54454E-01	9.89730E+02	2.48492E+01	1.12181E+03	3.42464E+01
	F10		F11		F12	
	AVG	STD	AVG	STD	AVG	STD
COWOA	2.03636E+03	4.51913E+02	4.93523E+03	6.08322E+02	**1.20032E+03**	2.20040E-01
ACWOA	4.62921E+03	8.29208E+02	6.40477E+03	1.04653E+03	1.20172E+03	5.38241E-01
IWOA	**1.46835E+03**	**2.11799E+02**	4.52086E+03	5.53069E+02	1.20042E+03	1.85634E-01
BMWOA	4.76675E+03	6.85060E+02	7.16619E+03	7.63418E+02	1.20243E+03	5.46247E-01
LWOA	2.04076E+03	4.02744E+02	5.46081E+03	7.82027E+02	1.20085E+03	2.75278E-01

EWOA	1.68352E+03	3.22534E+02	**4.48549E+03**	6.35311E+02	1.20041E+03	**1.79152E-01**
CWOA	5.19736E+03	6.76962E+02	6.38526E+03	5.74394E+02	1.20193E+03	4.69232E-01
OBWOA	4.74517E+03	7.43820E+02	6.04019E+03	**5.50887E+02**	1.20173E+03	4.53633E-01
	F13		F14		F15	
	AVG	STD	AVG	STD	AVG	STD
COWOA	1.30055E+03	1.25103E-01	1.40031E+03	5.41868E-02	1.53669E+03	1.80893E+01
ACWOA	1.30141E+03	9.42524E-01	1.41876E+03	1.21096E+01	2.13908E+03	6.37226E+02
IWOA	1.30052E+03	1.07572E-01	1.40032E+03	1.67887E-01	1.52747E+03	1.03679E+01
BMWOA	1.30061E+03	1.02999E-01	1.40033E+03	1.29443E-01	1.58602E+03	4.02737E+01
LWOA	1.30054E+03	1.30769E-01	**1.40028E+03**	**3.99044E-02**	1.52156E+03	**5.51242E+00**
EWOA	**1.30050E+03**	**1.00870E-01**	1.40029E+03	5.54070E-02	**1.51821E+03**	7.30373E+00
CWOA	1.30078E+03	6.10498E-01	1.40505E+03	8.50782E+00	2.14433E+03	7.48902E+02
OBWOA	1.30050E+03	1.06703E-01	1.40031E+03	1.60314E-01	1.62106E+03	9.40169E+01
	F16		F17		F18	
	AVG	STD	AVG	STD	AVG	STD
COWOA	1.61229E+03	6.04012E-01	**3.43146E+03**	**4.47137E+02**	**2.02009E+03**	**6.78690E+01**
ACWOA	1.61221E+03	4.71630E-01	1.44577E+07	8.83046E+06	5.01298E+07	3.15539E+07
IWOA	**1.61172E+03**	7.08852E-01	1.05751E+06	9.96746E+05	6.49683E+03	4.47855E+03
BMWOA	1.61250E+03	**2.60176E-01**	5.29388E+06	3.59030E+06	1.07226E+05	1.12769E+05
LWOA	1.61247E+03	5.53266E-01	4.69910E+05	2.53821E+05	8.92200E+03	3.81137E+03
EWOA	1.61177E+03	5.36307E-01	8.40468E+05	5.24889E+05	5.38143E+03	3.96062E+03
CWOA	1.61277E+03	4.05185E-01	6.58713E+06	5.78221E+06	8.39846E+06	2.47742E+07
OBWOA	1.61231E+03	3.65919E-01	8.34806E+06	2.45041E+07	3.33189E+08	1.26825E+09
	F19		F20		F21	
	AVG	STD	AVG	STD	AVG	STD
COWOA	**1.91222E+03**	**2.25471E+00**	**2.27329E+03**	**1.17261E+02**	**3.10483E+03**	**2.98172E+02**
ACWOA	2.01195E+03	3.59835E+01	4.08910E+04	1.92958E+04	4.93316E+06	4.38945E+06
IWOA	1.92817E+03	3.52522E+01	4.81797E+03	2.90398E+03	5.89679E+05	3.91308E+05
BMWOA	1.93066E+03	1.99677E+01	3.81167E+04	2.24651E+04	1.42878E+06	9.51375E+05
LWOA	1.91805E+03	1.76018E+01	3.10424E+03	7.11592E+02	1.97185E+05	1.36415E+05
EWOA	1.92455E+03	3.22555E+01	3.49621E+03	2.71621E+03	3.29482E+05	2.82118E+05
CWOA	1.99332E+03	4.08384E+01	5.45102E+04	3.18236E+04	4.37175E+06	3.40550E+06
OBWOA	1.95486E+03	4.94046E+01	2.46746E+04	1.05621E+04	4.93091E+06	1.60316E+07
	F22		F23		F24	
	AVG	STD	AVG	STD	AVG	STD
COWOA	2.82501E+03	2.19678E+02	2.61401E+03	**0.00000E+00**	2.60082E+03	4.47475E+00
ACWOA	3.04673E+03	2.59867E+02	2.50686E+03	3.75791E+01	**2.60000E+03**	**2.67265E-06**
IWOA	2.78805E+03	1.93856E+02	2.61536E+03	2.66148E-01	2.60204E+03	6.89157E+00
BMWOA	3.02884E+03	2.46317E+02	2.50040E+03	4.23767E-01	2.60021E+03	1.94689E-01
LWOA	2.93930E+03	1.89542E+02	2.61546E+03	1.26984E-01	2.60680E+03	8.83798E+00
EWOA	**2.70642E+03**	**1.58871E+02**	2.61531E+03	2.73385E-01	2.60544E+03	1.12814E+01
CWOA	3.03760E+03	3.54810E+02	2.64705E+03	3.17564E+01	2.60588E+03	5.54547E+00
OBWOA	3.13229E+03	7.74074E+02	**2.50000E+03**	2.80071E-13	2.60106E+03	8.05403E-01
	F25		F26		F27	
	AVG	STD	AVG	STD	AVG	STD
COWOA	2.70948E+03	1.08506E+01	2.70382E+03	1.81685E+01	3.68321E+03	2.17173E+02
ACWOA	**2.70000E+03**	**0.00000E+00**	2.74719E+03	5.02454E+01	3.62417E+03	3.66336E+02
IWOA	2.71198E+03	9.82671E+00	2.70054E+03	1.43104E-01	3.47279E+03	2.94223E+02
BMWOA	2.70001E+03	5.32118E-03	2.70058E+03	1.28700E-01	**2.90014E+03**	**1.92402E-01**
LWOA	2.71882E+03	9.74014E+00	**2.70054E+03**	**1.20325E-01**	3.71909E+03	3.26159E+02
EWOA	2.71501E+03	8.59932E+00	2.72912E+03	5.96357E+01	3.57324E+03	2.54256E+02
CWOA	2.70868E+03	1.38155E+01	2.73370E+03	4.76794E+01	3.76801E+03	3.61981E+02
OBWOA	2.70000E+03	2.53333E-13	2.78154E+03	3.65209E+01	2.91117E+03	6.11578E+01
	F28		F29		F30	
	AVG	STD	AVG	STD	AVG	STD
COWOA	4.45097E+03	4.79383E+02	**3.20172E+03**	**1.59904E+02**	**3.87907E+03**	**2.79275E+02**
ACWOA	4.00712E+03	1.20064E+03	1.76982E+07	1.50452E+07	3.92035E+05	3.23887E+05

IWOA	4.33373E+03	3.93017E+02	3.69382E+06	4.29284E+06	9.12362E+03	2.70570E+03
BMWOA	**3.00014E+03**	**2.03061E-01**	7.14824E+05	6.97189E+05	4.32653E+04	3.66914E+04
LWOA	4.77153E+03	5.94597E+02	6.45160E+06	3.95442E+06	1.09509E+04	4.45497E+03
EWOA	4.36658E+03	4.44038E+02	5.25202E+06	4.36187E+06	1.10419E+04	6.28527E+03
CWOA	5.23933E+03	9.35767E+02	6.77828E+06	4.75826E+06	1.34901E+05	9.69893E+04
OBWOA	3.70925E+03	1.24061E+03	2.07036E+06	4.24048E+06	9.33839E+03	2.03051E+04

Table A.3 Comparison results of COWOA with the well-known original algorithm

	F1		F2		F3	
	AVG	STD	AVG	STD	AVG	STD
COWOA	**1.00000E+02**	**2.82278E-08**	**2.00000E+02**	**1.58333E-14**	**3.00000E+02**	**2.79274E-14**
SCA	2.47893E+08	8.26677E+07	1.66492E+10	2.43198E+09	3.70902E+04	6.15293E+03
GWO	5.44238E+07	4.07192E+07	2.50874E+09	2.47941E+09	3.12685E+04	8.96277E+03
MFO	1.05537E+08	1.09583E+08	1.29754E+10	7.04051E+09	1.00644E+05	4.22552E+04
GOA	1.23077E+07	9.00918E+06	2.27158E+07	1.24337E+08	7.04537E+03	8.31355E+03
BA	8.09696E+05	4.44046E+05	6.22503E+05	2.79844E+05	4.71483E+02	2.90893E+02
PSO	8.50982E+06	2.25858E+06	1.49781E+08	1.52868E+07	9.46843E+02	1.26169E+02
CSA	1.98423E+06	1.25484E+06	1.14868E+04	8.73514E+03	9.87165E+02	3.75723E+02
FA	2.72201E+08	3.51568E+07	1.55474E+10	1.82530E+09	6.27729E+04	1.00122E+04
ACOR	7.67000E+06	9.41883E+06	8.26737E+07	2.83258E+08	1.08023E+04	1.38328E+04
	F4		F5		F6	
	AVG	STD	AVG	STD	AVG	STD
COWOA	**4.00785E+02**	**1.64296E+00**	5.20000E+02	**1.12811E-05**	6.25708E+02	3.43137E+00
SCA	1.33191E+03	1.81518E+02	5.20939E+02	5.67892E-02	6.33787E+02	2.23624E+00
GWO	6.53805E+02	7.52869E+01	5.20954E+02	4.81634E-02	6.14257E+02	2.41666E+00
MFO	1.68381E+03	1.68844E+03	5.20293E+02	1.71570E-01	6.24087E+02	4.07186E+00
GOA	5.26197E+02	3.70677E+01	5.20114E+02	9.63179E-02	6.18139E+02	3.73104E+00
BA	4.34448E+02	3.91227E+01	5.20964E+02	3.18181E-02	6.33713E+02	3.74399E+00
PSO	4.74263E+02	4.02354E+01	5.20960E+02	4.14936E-02	6.22849E+02	3.98922E+00
CSA	5.34822E+02	6.91638E+01	**5.20000E+02**	6.19853E-04	6.30093E+02	3.77343E+00
FA	1.59216E+03	1.77753E+02	5.20945E+02	5.32769E-02	6.33899E+02	**8.89601E-01**
ACOR	4.79238E+02	5.73196E+01	5.20927E+02	4.87391E-02	**6.13505E+02**	3.75418E+00
	F7		F8		F9	
	AVG	STD	AVG	STD	AVG	STD
COWOA	**7.00000E+02**	**7.89906E-14**	8.78635E+02	1.98793E+01	1.06135E+03	2.86225E+01
SCA	8.37396E+02	2.93990E+01	1.03753E+03	1.82823E+01	1.16967E+03	1.61883E+01
GWO	7.18308E+02	1.44643E+01	8.75324E+02	1.52571E+01	**1.00458E+03**	2.38696E+01
MFO	8.14075E+02	7.33520E+01	9.47429E+02	3.99056E+01	1.10845E+03	4.62508E+01
GOA	7.02391E+02	4.97139E+00	9.07298E+02	3.18401E+01	1.01383E+03	2.64065E+01
BA	7.00589E+02	1.96788E-01	1.02765E+03	4.82522E+01	1.18761E+03	6.54014E+01
PSO	7.02331E+02	1.30560E-01	9.70004E+02	1.85997E+01	1.11407E+03	3.14784E+01
CSA	7.00009E+02	6.72208E-03	9.32628E+02	2.21604E+01	1.05515E+03	2.22476E+01
FA	8.35960E+02	1.19303E+01	1.02142E+03	**1.50608E+01**	1.16153E+03	**1.39574E+01**
ACOR	7.05110E+02	1.41997E+01	**8.61887E+02**	1.70349E+01	1.02716E+03	6.51847E+01
	F10		F11		F12	
	AVG	STD	AVG	STD	AVG	STD
COWOA	**2.10840E+03**	3.84666E+02	4.86711E+03	6.03830E+02	**1.20026E+03**	**1.42313E-01**
SCA	6.86082E+03	5.78155E+02	8.06434E+03	**3.23267E+02**	1.20247E+03	2.87203E-01
GWO	2.99253E+03	4.59747E+02	**4.06664E+03**	7.16286E+02	1.20132E+03	1.16887E+00
MFO	4.57641E+03	8.92699E+02	5.41724E+03	9.41295E+02	1.20053E+03	3.07008E-01
GOA	4.71571E+03	6.45989E+02	4.76012E+03	8.11356E+02	1.20066E+03	4.00249E-01
BA	5.26596E+03	6.67114E+02	5.77133E+03	6.72117E+02	1.20130E+03	3.50209E-01

PSO	5.10593E+03	4.86141E+02	5.89851E+03	6.30384E+02	1.20243E+03	3.37968E-01
CSA	4.45269E+03	7.29746E+02	5.11948E+03	6.31093E+02	1.20086E+03	2.86410E-01
FA	7.53190E+03	**2.75981E+02**	7.88181E+03	3.25708E+02	1.20253E+03	2.73255E-01
ACOR	3.06111E+03	5.30241E+02	4.96787E+03	2.17430E+03	1.20227E+03	4.36353E-01
	F13		F14		F15	
	AVG	STD	AVG	STD	AVG	STD
COWOA	1.30052E+03	1.04362E-01	1.40036E+03	1.83866E-01	1.53451E+03	2.15206E+01
SCA	1.30292E+03	2.54095E-01	1.44150E+03	7.32182E+00	4.18059E+03	2.39719E+03
GWO	1.30048E+03	3.99652E-01	1.40384E+03	6.11808E+00	1.76331E+03	6.07950E+02
MFO	1.30170E+03	1.07965E+00	1.43542E+03	2.47940E+01	2.67277E+05	6.77262E+05
GOA	1.30043E+03	1.31347E-01	1.40057E+03	3.70083E-01	**1.50789E+03**	2.20664E+00
BA	1.30048E+03	1.20851E-01	1.40031E+03	1.05753E-01	1.52849E+03	5.17127E+00
PSO	**1.30038E+03**	**9.08694E-02**	**1.40027E+03**	8.53407E-02	1.51692E+03	**1.13823E+00**
CSA	1.30050E+03	1.14358E-01	1.40027E+03	**4.65959E-02**	1.52614E+03	1.31447E+01
FA	1.30276E+03	2.33500E-01	1.43935E+03	5.51451E+00	1.48853E+04	4.84787E+03
ACOR	1.30051E+03	1.11462E-01	1.40063E+03	2.60457E-01	1.60066E+03	1.87702E+02
	F16		F17		F18	
	AVG	STD	AVG	STD	AVG	STD
COWOA	1.61228E+03	6.14302E-01	**3.39821E+03**	**5.01096E+02**	**2.04300E+03**	**6.74184E+01**
SCA	1.61284E+03	2.68096E-01	5.86327E+06	1.94758E+06	1.65592E+08	8.60196E+07
GWO	**1.61099E+03**	6.59545E-01	1.72920E+06	2.03433E+06	1.01135E+07	2.17959E+07
MFO	1.61268E+03	5.65559E-01	2.70502E+06	4.42109E+06	1.75013E+07	6.54528E+07
GOA	1.61178E+03	5.13383E-01	3.20387E+05	3.13498E+05	7.85458E+03	5.94705E+03
BA	1.61336E+03	3.77671E-01	1.06061E+05	5.77302E+04	8.82978E+04	4.20742E+04
PSO	1.61196E+03	4.19728E-01	2.40599E+05	1.23321E+05	2.19847E+06	5.70553E+05
CSA	1.61222E+03	4.37397E-01	1.90552E+04	1.18682E+04	2.13877E+03	8.99417E+01
FA	1.61284E+03	**2.30407E-01**	6.95172E+06	1.92838E+06	2.85790E+08	9.37674E+07
ACOR	1.61164E+03	3.62039E-01	1.88897E+05	3.09770E+05	4.68859E+04	2.13766E+05
	F19		F20		F21	
	AVG	STD	AVG	STD	AVG	STD
COWOA	**1.91241E+03**	4.84369E+00	**2.23106E+03**	**7.82369E+01**	**3.05617E+03**	**2.93844E+02**
SCA	1.98554E+03	1.89515E+01	1.76514E+04	6.84819E+03	1.50498E+06	4.93611E+05
GWO	1.95016E+03	2.92615E+01	1.66837E+04	9.97919E+03	7.06011E+05	9.41847E+05
MFO	1.97055E+03	5.16599E+01	6.47283E+04	3.49499E+04	6.88109E+05	1.06451E+06
GOA	1.91406E+03	2.08496E+00	3.95171E+03	7.62006E+03	1.24167E+05	1.30592E+05
BA	1.92434E+03	2.23534E+01	2.40131E+03	1.19355E+02	5.40835E+04	3.27190E+04
PSO	1.91633E+03	**2.07927E+00**	2.33914E+03	7.90848E+01	9.47002E+04	5.82891E+04
CSA	1.93087E+03	2.30656E+01	2.40462E+03	1.59498E+02	1.28138E+04	4.10788E+03
FA	2.00743E+03	1.46898E+01	1.86877E+04	6.59675E+03	1.70864E+06	7.54411E+05
ACOR	1.92519E+03	3.12647E+01	9.21813E+03	1.97438E+04	8.13799E+04	6.63327E+04
	F22		F23		F24	
	AVG	STD	AVG	STD	AVG	STD
COWOA	2.79475E+03	2.06453E+02	**2.61401E+03**	**0.00000E+00**	2.60197E+03	7.33594E+00
SCA	2.90690E+03	2.04340E+02	2.66412E+03	8.60396E+00	2.60008E+03	9.61257E-02
GWO	2.58913E+03	1.73143E+02	2.63340E+03	7.83387E+00	**2.60000E+03**	**3.79612E-04**
MFO	2.98790E+03	2.42083E+02	2.67876E+03	4.32175E+01	2.66937E+03	3.13665E+01
GOA	2.65985E+03	1.62126E+02	2.62666E+03	1.08386E+01	2.63647E+03	7.40319E+00
BA	3.29740E+03	3.12199E+02	2.61525E+03	2.25457E-03	2.66208E+03	1.77883E+01
PSO	2.87841E+03	2.04276E+02	2.61601E+03	4.58025E-01	2.62515E+03	5.64883E+00
CSA	2.83132E+03	2.61260E+02	2.61670E+03	1.08782E+00	2.61526E+03	1.27440E+01
FA	2.92889E+03	**1.42502E+02**	2.73691E+03	1.77816E+01	2.70510E+03	5.54628E+00

ACOR	**2.56305E+03**	2.25484E+02	2.61769E+03	4.67342E+00	2.64289E+03	7.20875E+00
	F25		F26		F27	
	AVG	STD	AVG	STD	AVG	STD
COWOA	2.71465E+03	1.07497E+01	2.70713E+03	2.52442E+01	3.56651E+03	2.99048E+02
SCA	2.72489E+03	1.03468E+01	2.70217E+03	4.88831E-01	3.62925E+03	3.34981E+02
GWO	2.71074E+03	3.74955E+00	2.74692E+03	5.05136E+01	3.35927E+03	1.17634E+02
MFO	2.71775E+03	9.33137E+00	2.70256E+03	1.46317E+00	3.65364E+03	2.04362E+02
GOA	2.70823E+03	**2.45879E+00**	2.76268E+03	6.72447E+01	3.44426E+03	1.61926E+02
BA	2.72666E+03	8.92416E+00	2.70049E+03	1.59513E-01	3.93086E+03	3.47100E+02
PSO	2.71304E+03	6.59502E+00	2.78713E+03	3.45627E+01	3.33674E+03	2.59929E+02
CSA	2.70929E+03	3.24367E+00	**2.70044E+03**	**9.53737E-02**	**3.17891E+03**	2.25544E+02
FA	2.73446E+03	4.97346E+00	2.70228E+03	2.44614E-01	3.77488E+03	**8.21619E+01**
ACOR	**2.70747E+03**	2.92006E+00	2.72843E+03	7.57776E+01	3.41502E+03	8.62024E+01
	F28		F29		F30	
	AVG	STD	AVG	STD	AVG	STD
COWOA	4.35845E+03	4.94617E+02	**3.22982E+03**	**1.95945E+02**	**3.96814E+03**	**2.18466E+02**
SCA	4.89625E+03	3.60381E+02	1.13756E+07	6.90465E+06	2.58054E+05	9.03785E+04
GWO	3.99628E+03	3.44513E+02	4.82035E+05	1.05929E+06	6.09713E+04	4.25557E+04
MFO	3.95725E+03	2.50766E+02	2.09424E+06	3.36497E+06	4.60470E+04	4.29122E+04
GOA	4.16834E+03	4.15597E+02	4.31094E+06	8.70571E+06	2.83061E+04	3.00813E+04
BA	5.34803E+03	7.08830E+02	3.70409E+07	4.63416E+07	2.53831E+04	4.95725E+04
PSO	6.94157E+03	1.12089E+03	4.94286E+04	9.37238E+04	1.36847E+04	6.21954E+03
CSA	6.50178E+03	7.00447E+02	2.27970E+06	1.24534E+07	1.06095E+04	2.84019E+03
FA	4.28294E+03	2.33714E+02	3.18340E+06	1.28348E+06	1.72718E+05	4.26586E+04
ACOR	**3.89983E+03**	**2.23007E+02**	1.96184E+06	3.99633E+06	9.14425E+03	3.81024E+03

Table A.4 Comparison results of COWOA with the well-known variant algorithms

	F1		F2		F3	
	AVG	STD	AVG	STD	AVG	STD
COWOA	**1.00000E+02**	**1.17474E-08**	**2.00000E+02**	**1.49278E-14**	**3.00000E+02**	**3.94953E-14**
SCAPSO	8.35826E+06	3.20892E+06	3.60625E+07	8.65431E+06	2.93145E+03	1.00910E+03
RCBA	1.18646E+06	5.45937E+05	2.62186E+04	1.22716E+04	3.23929E+02	7.36027E+00
CBA	4.46386E+06	1.21536E+06	8.67395E+03	9.73275E+03	5.32496E+03	6.66802E+03
HGWO	1.91740E+08	4.28345E+07	8.50732E+09	1.83313E+09	6.59416E+04	8.70869E+03
OBLGWO	1.94926E+07	9.80254E+06	1.41726E+07	1.05013E+07	9.36015E+03	3.17761E+03
mSCA	6.42685E+07	4.45273E+07	5.71893E+09	3.65135E+09	2.54945E+04	7.56570E+03
CDLOBA	7.10694E+05	4.78510E+05	1.04415E+04	1.03705E+04	7.34862E+04	2.32060E+04
CAGWO	4.76509E+07	2.20792E+07	3.62173E+08	2.91824E+08	2.77372E+04	7.73461E+03
	F4		F5		F6	
	AVG	STD	AVG	STD	AVG	STD
COWOA	**4.00883E+02**	**1.47735E+00**	**5.20000E+02**	**4.00012E-06**	6.25740E+02	3.66744E+00
SCAPSO	4.72692E+02	3.38501E+01	5.20946E+02	5.67497E-02	6.29310E+02	3.28591E+00
RCBA	4.75496E+02	3.60434E+01	5.20069E+02	7.44775E-02	6.38830E+02	4.02305E+00
CBA	4.98165E+02	3.93021E+01	5.20197E+02	2.12350E-01	6.40878E+02	3.12738E+00
HGWO	9.21329E+02	6.67359E+01	5.20857E+02	1.28810E-01	6.25946E+02	**1.69618E+00**
OBLGWO	5.38811E+02	3.98128E+01	5.20955E+02	4.60968E-02	6.20749E+02	5.03876E+00
mSCA	7.77284E+02	1.45251E+02	5.20596E+02	1.61283E-01	6.21269E+02	2.83919E+00
CDLOBA	4.83688E+02	4.89755E+01	5.20862E+02	2.35291E-01	6.35788E+02	2.56364E+00
CAGWO	5.80309E+02	3.86702E+01	5.21028E+02	5.52928E-02	**6.11272E+02**	2.67817E+00
	F7		F8		F9	
	AVG	STD	AVG	STD	AVG	STD

COWOA	**7.00001E+02**	**2.25674E-03**	**8.81487E+02**	1.95884E+01	1.06211E+03	3.30183E+01
SCAPSO	7.01351E+02	9.14333E-02	9.85630E+02	2.97762E+01	1.11168E+03	3.10327E+01
RCBA	7.00071E+02	2.01926E-02	1.02723E+03	5.44564E+01	1.14688E+03	5.12973E+01
CBA	7.00014E+02	1.47003E-02	1.03684E+03	4.49053E+01	1.16483E+03	6.02832E+01
HGWO	7.43264E+02	9.23860E+00	1.00972E+03	**1.26018E+01**	1.13703E+03	**1.66615E+01**
OBLGWO	7.01173E+02	8.40152E-02	9.23651E+02	2.31727E+01	1.07036E+03	3.76222E+01
mSCA	7.54703E+02	3.70076E+01	9.35237E+02	2.52730E+01	1.05697E+03	2.05405E+01
CDLOBA	7.00010E+02	9.10703E-03	1.06086E+03	3.84640E+01	1.24742E+03	6.43062E+01
CAGWO	7.05650E+02	5.64127E+00	8.96102E+02	4.70100E+01	**1.03198E+03**	5.21167E+01
	F10		F11		F12	
	AVG	STD	AVG	STD	AVG	STD
COWOA	**1.99026E+03**	4.92474E+02	4.96256E+03	8.11459E+02	**1.20027E+03**	**1.88270E-01**
SCAPSO	5.30912E+03	8.11478E+02	5.82343E+03	6.79223E+02	1.20202E+03	3.43861E-01
RCBA	5.63942E+03	6.01052E+02	5.57137E+03	6.09533E+02	1.20064E+03	3.69055E-01
CBA	5.66791E+03	6.94660E+02	5.77267E+03	6.05625E+02	1.20106E+03	4.45535E-01
HGWO	5.69093E+03	**3.24414E+02**	6.56070E+03	**5.77692E+02**	1.20136E+03	3.32246E-01
OBLGWO	3.94470E+03	9.33003E+02	5.39168E+03	9.52352E+02	1.20245E+03	5.01621E-01
mSCA	4.16190E+03	6.39927E+02	**4.90057E+03**	7.11229E+02	1.20071E+03	3.53396E-01
CDLOBA	5.42683E+03	7.01060E+02	5.66067E+03	6.64156E+02	1.20040E+03	2.64744E-01
CAGWO	4.97265E+03	1.88841E+03	6.97179E+03	1.50472E+03	1.20298E+03	3.51644E-01
	F13		F14		F15	
	AVG	STD	AVG	STD	AVG	STD
COWOA	1.30049E+03	1.06802E-01	1.40031E+03	1.27175E-01	1.53193E+03	1.98577E+01
SCAPSO	1.30044E+03	1.12714E-01	**1.40023E+03**	**4.49324E-02**	1.51691E+03	**1.40121E+00**
RCBA	1.30049E+03	1.14130E-01	1.40029E+03	8.64743E-02	1.53691E+03	1.03483E+01
CBA	1.30052E+03	1.61165E-01	1.40034E+03	1.42464E-01	1.56553E+03	1.47782E+01
HGWO	1.30203E+03	3.24506E-01	1.42164E+03	3.67320E+00	1.93766E+03	4.66411E+02
OBLGWO	1.30052E+03	1.30390E-01	1.40044E+03	2.00399E-01	**1.51574E+03**	4.82831E+00
mSCA	1.30091E+03	6.15067E-01	1.41359E+03	7.81908E+00	2.44850E+03	1.24052E+03
CDLOBA	1.30046E+03	1.16078E-01	1.40030E+03	5.25399E-02	1.71841E+03	7.12114E+01
CAGWO	**1.30038E+03**	**7.33607E-02**	1.40041E+03	1.00516E-01	1.52002E+03	6.76316E+00
	F16		F17		F18	
	AVG	STD	AVG	STD	AVG	STD
COWOA	1.61224E+03	5.61031E-01	**3.38123E+03**	**3.76039E+02**	**2.03132E+03**	**6.61410E+01**
SCAPSO	1.61242E+03	4.12689E-01	2.11628E+05	1.44953E+05	6.91929E+05	3.25283E+05
RCBA	1.61335E+03	3.85061E-01	1.44238E+05	7.51419E+04	1.28911E+04	1.20854E+04
CBA	1.61335E+03	**2.60882E-01**	2.17275E+05	1.29869E+05	6.36956E+03	5.55354E+03
HGWO	1.61257E+03	2.96830E-01	5.09351E+06	2.54180E+06	1.21301E+08	3.18976E+07
OBLGWO	1.61199E+03	5.39014E-01	1.12575E+06	9.37116E+05	4.97823E+04	7.16981E+04
mSCA	**1.61177E+03**	5.22336E-01	1.76755E+06	1.54434E+06	1.57308E+07	3.78756E+07
CDLOBA	1.61337E+03	3.39588E-01	3.80060E+04	3.42077E+04	8.93285E+03	8.25561E+03
CAGWO	1.61190E+03	2.69899E-01	1.29935E+06	9.58223E+05	4.49744E+05	7.10857E+05
	F19		F20		F21	
	AVG	STD	AVG	STD	AVG	STD
COWOA	**1.91226E+03**	**2.85537E+00**	**2.22435E+03**	**9.32814E+01**	**3.08494E+03**	**2.76748E+02**
SCAPSO	1.91857E+03	3.16047E+00	2.46059E+03	2.18025E+02	7.12671E+04	3.58202E+04
RCBA	1.93020E+03	3.19310E+01	2.37917E+03	1.01573E+02	6.24062E+04	3.90759E+04
CBA	1.93069E+03	2.50142E+01	4.00101E+03	2.25494E+03	1.13512E+05	5.92755E+04
HGWO	1.99186E+03	9.09114E+00	5.91070E+04	2.36770E+04	2.84159E+06	2.69220E+06
OBLGWO	1.91685E+03	1.78574E+01	6.01463E+03	2.78893E+03	6.20113E+05	3.96766E+05
mSCA	1.94516E+03	2.37416E+01	1.14140E+04	5.34070E+03	5.45678E+05	4.14070E+05

CDLOBA	1.96923E+03	3.54070E+01	3.01354E+04	1.68017E+04	3.20698E+04	1.74151E+04
CAGWO	1.92810E+03	6.83075E+00	2.19102E+04	5.12507E+03	2.80693E+05	2.28928E+05
	F22		F23		F24	
	AVG	STD	AVG	STD	AVG	STD
COWOA	2.74444E+03	2.35682E+02	2.61401E+03	**0.00000E+00**	2.60338E+03	8.84224E+00
SCAPSO	3.10240E+03	2.97956E+02	**2.50000E+03**	0.00000E+00	**2.60000E+03**	**0.00000E+00**
RCBA	3.38379E+03	3.53335E+02	2.61525E+03	5.44647E-03	2.68380E+03	3.21517E+01
CBA	3.48199E+03	3.40470E+02	2.61585E+03	3.58140E-01	2.66393E+03	2.89573E+01
HGWO	2.98297E+03	**1.59399E+02**	2.52834E+03	6.45575E+01	2.60000E+03	4.73266E-12
OBLGWO	2.72212E+03	1.69649E+02	2.61434E+03	2.17189E+01	2.60545E+03	1.44242E+01
m SCA	2.59619E+03	1.97612E+02	2.63737E+03	7.73319E+00	2.60000E+03	6.75370E-04
CDLOBA	3.28116E+03	2.31598E+02	2.61599E+03	2.18938E+00	2.69749E+03	4.57342E+01
CAGWO	**2.57018E+03**	1.71108E+02	2.62615E+03	3.85768E+00	2.60000E+03	7.53347E-05
	F25		F26		F27	
	AVG	STD	AVG	STD	AVG	STD
COWOA	2.71052E+03	1.02209E+01	**2.70047E+03**	**1.28100E-01**	3.62486E+03	2.85788E+02
SCAPSO	**2.70000E+03**	**0.00000E+00**	2.77345E+03	4.47746E+01	**2.90000E+03**	**0.00000E+00**
RCBA	2.73138E+03	1.51011E+01	2.73782E+03	9.47068E+01	3.93736E+03	4.32443E+02
CBA	2.73867E+03	2.07107E+01	2.73179E+03	9.56113E+01	4.04351E+03	3.29751E+02
HGWO	2.70000E+03	0.00000E+00	2.73487E+03	4.68894E+01	3.65025E+03	1.57396E+02
OBLGWO	2.70000E+03	0.00000E+00	2.70054E+03	1.54108E-01	2.96694E+03	2.07617E+02
mSCA	2.71195E+03	3.80289E+00	2.70079E+03	1.99257E-01	3.17752E+03	1.18424E+02
CDLOBA	2.72166E+03	1.27351E+01	2.72439E+03	7.81286E+01	3.75988E+03	4.70363E+02
CAGWO	2.70000E+03	0.00000E+00	2.73036E+03	4.39839E+01	3.24401E+03	5.51140E+01
	F28		F29		F30	
	AVG	STD	AVG	STD	AVG	STD
COWOA	4.41826E+03	5.94356E+02	3.24556E+03	2.01036E+02	**3.92115E+03**	**2.83929E+02**
SCAPSO	**3.00000E+03**	**0.00000E+00**	**3.11311E+03**	**1.10590E+01**	1.26964E+04	8.03237E+03
RCBA	5.71203E+03	1.10747E+03	1.88649E+07	1.26193E+07	1.14605E+04	8.23581E+03
CBA	5.47031E+03	8.22564E+02	2.75641E+07	3.18884E+07	3.80577E+04	8.70656E+04
HGWO	4.26079E+03	2.45228E+02	3.16185E+06	3.06619E+06	1.26821E+04	5.19354E+04
OBLGWO	3.58782E+03	5.66337E+02	4.92848E+06	4.36520E+06	2.55296E+04	2.31112E+04
mSCA	3.90969E+03	2.29183E+02	2.04363E+06	4.58191E+06	4.83060E+04	2.19918E+04
CDLOBA	5.39895E+03	7.84669E+02	1.13210E+07	1.01920E+07	4.45229E+04	7.66823E+04

Table A.5 Comparison results of the Accuracy

**Dataset**	Breast		Ionosphere		HeartEW	
**Metric**	Avg	Std	Avg	Std	Avg	Std
bCOWOA	**9.89470E-01**	1.47960E-02	**9.71650E-01**	2.62010E-02	**9.48150E-01**	3.98140E-02
bWOA	9.85900E-01	1.12300E-02	9.54350E-01	4.09220E-02	9.29630E-01	4.76550E-02
bGWO	9.59550E-01	2.97320E-02	8.74780E-01	4.21160E-02	8.29630E-01	7.84700E-02
bHHO	9.87720E-01	1.18410E-02	9.62940E-01	2.71340E-02	9.25930E-01	4.27670E-02
bMFO	9.87720E-01	1.44500E-02	9.63010E-01	2.71820E-02	9.33330E-01	**3.40350E-02**
bSCA	9.87690E-01	2.04770E-02	9.54360E-01	3.07770E-02	9.33330E-01	4.20480E-02
bSMA	9.87660E-01	1.86710E-02	9.68630E-01	2.82870E-02	9.40740E-01	3.98140E-02
bSSA	9.84150E-01	**1.00960E-02**	9.62850E-01	**2.35230E-02**	9.40740E-01	6.80690E-02
bCSO	9.85900E-01	1.62140E-02	9.57110E-01	2.78300E-02	9.33330E-01	5.46570E-02
BBA	9.38590E-01	4.07310E-02	8.77420E-01	4.97030E-02	8.22220E-01	8.33680E-02
**Dataset**	Congress		Breastcancer		heart	
**Metric**	Avg	Std	Avg	Std	Avg	Std
bCOWOA	**9.77060E-01**	1.87040E-02	**9.88570E-01**	1.31280E-02	**9.48150E-01**	3.98140E-02

bWOA	9.76900E-01	2.44050E-02	9.87100E-01	1.06100E-02	9.37040E-01	3.92350E-02
bGWO	9.37790E-01	3.78740E-02	9.57100E-01	2.42540E-02	8.37040E-01	7.02730E-02
bHHO	9.72300E-01	2.85330E-02	9.85690E-01	**9.52450E-03**	9.37040E-01	5.80380E-02
bMFO	9.72360E-01	2.11830E-02	9.87120E-01	1.83830E-02	9.37040E-01	3.04920E-02
bSCA	9.74630E-01	1.70360E-02	9.85730E-01	1.15010E-02	9.40740E-01	3.12320E-02
bSMA	9.72410E-01	1.81530E-02	9.84290E-01	1.42060E-02	9.37040E-01	**2.49980E-02**
bSSA	9.70030E-01	**1.56570E-02**	9.85710E-01	1.34690E-02	9.29630E-01	3.24290E-02
bCSO	9.72460E-01	2.60650E-02	9.87140E-01	1.25120E-02	9.37040E-01	4.29450E-02
BBA	8.63880E-01	9.22130E-02	9.55710E-01	2.56010E-02	8.11110E-01	1.22780E-01

Table A.6 Comparison results of the Precision

**Dataset**	Breast		Ionosphere		HeartEW	
**Metric**	Avg	Std	Avg	Std	Avg	Std
bCOWOA	**9.83850E-01**	2.25740E-02	**9.58900E-01**	3.67000E-02	**9.49170E-01**	4.95660E-02
bWOA	9.81010E-01	1.82800E-02	9.36800E-01	5.44550E-02	9.25670E-01	5.54860E-02
bGWO	9.52510E-01	3.76690E-02	8.53770E-01	5.31870E-02	8.33530E-01	9.48700E-02
bHHO	9.83850E-01	1.86340E-02	9.47100E-01	3.70080E-02	9.22210E-01	**2.57780E-02**
bMFO	9.81220E-01	2.20040E-02	9.50450E-01	3.68290E-02	9.42440E-01	4.64090E-02
bSCA	9.81580E-01	3.05130E-02	9.39280E-01	3.96360E-02	9.24800E-01	4.85810E-02
bSMA	9.81500E-01	2.76410E-02	9.54950E-01	3.94450E-02	9.37020E-01	4.57170E-02
bSSA	9.75530E-01	**1.53740E-02**	9.46120E-01	**3.26540E-02**	9.31940E-01	7.29180E-02
bCSO	9.78660E-01	2.44270E-02	9.41960E-01	3.35690E-02	9.31030E-01	5.93500E-02
BBA	9.30690E-01	4.81490E-02	8.59770E-01	4.75490E-02	8.33160E-01	6.83070E-02
**Dataset**	Congress		Breastcancer		heart	
**Metric**	Avg	Std	Avg	Std	Avg	Std
bCOWOA	**9.96430E-01**	1.12940E-02	**9.95700E-01**	**9.06920E-03**	**9.33650E-01**	5.71410E-02
bWOA	9.89020E-01	1.76810E-02	9.93520E-01	1.04330E-02	9.25640E-01	3.88710E-02
bGWO	9.69190E-01	3.53810E-02	9.71760E-01	2.27080E-02	8.36230E-01	7.03920E-02
bHHO	9.92300E-01	1.62560E-02	9.91350E-01	1.11670E-02	9.12770E-01	7.49020E-02
bMFO	9.96430E-01	**1.12940E-02**	9.93560E-01	1.42910E-02	9.17720E-01	5.93500E-02
bSCA	9.82140E-01	2.52540E-02	9.93520E-01	1.04330E-02	9.30320E-01	**3.40060E-02**
bSMA	9.92590E-01	1.56160E-02	9.93570E-01	1.44150E-02	9.20380E-01	3.83520E-02
bSSA	9.85450E-01	2.52290E-02	9.93520E-01	1.04270E-02	9.14980E-01	4.82820E-02
bCSO	9.92720E-01	1.53400E-02	9.91480E-01	1.47470E-02	9.15590E-01	5.37700E-02
BBA	8.98340E-01	8.47580E-02	9.67740E-01	2.65710E-02	8.01490E-01	1.13380E-01

Table A.7 Comparison results of the F-measure

**Dataset**	Breast		Ionosphere		HeartEW	
**Metric**	Avg	Std	Avg	Std	Avg	Std
bCOWOA	**9.91740E-01**	1.15760E-02	**9.78690E-01**	1.93600E-02	**9.53960E-01**	3.47770E-02
bWOA	9.88890E-01	8.78890E-03	9.66630E-01	2.91740E-02	9.37260E-01	4.45530E-02
bGWO	9.68590E-01	2.28850E-02	9.09970E-01	2.68140E-02	8.52880E-01	6.47020E-02
bHHO	9.90370E-01	9.20720E-03	9.72500E-01	1.95500E-02	9.33230E-01	4.08280E-02
bMFO	9.90410E-01	1.12810E-02	9.72050E-01	2.04490E-02	9.39920E-01	**3.05110E-02**
bSCA	9.90490E-01	1.57920E-02	9.66070E-01	2.16330E-02	9.41260E-01	3.74260E-02
bSMA	9.90480E-01	1.43030E-02	9.76580E-01	2.08020E-02	9.47450E-01	3.52860E-02
bSSA	9.87560E-01	**7.87460E-03**	9.72050E-01	**1.74160E-02**	9.48580E-01	5.87700E-02
bCSO	9.89080E-01	1.25380E-02	9.67800E-01	2.05260E-02	9.40740E-01	4.98300E-02
BBA	9.52620E-01	3.14280E-02	9.10430E-01	3.61310E-02	8.40000E-01	7.74450E-02
**Dataset**	Congress		Breastcancer		heart	
**Metric**	Avg	Std	Avg	Std	Avg	Std

bCOWOA	**9.80760E-01**	1.59230E-02	**9.91210E-01**	1.01300E-02	**9.55190E-01**	3.36840E-02
bWOA	9.80580E-01	2.10610E-02	9.90110E-01	8.18340E-03	9.43700E-01	3.72430E-02
bGWO	9.47870E-01	3.17620E-02	9.67000E-01	1.88630E-02	8.57520E-01	6.25200E-02
bHHO	9.76980E-01	2.36720E-02	9.89060E-01	**7.24750E-03**	9.47110E-01	4.80130E-02
bMFO	9.76670E-01	1.84890E-02	9.90110E-01	1.42090E-02	9.46080E-01	2.40590E-02
bSCA	9.79150E-01	1.42800E-02	9.89010E-01	8.97390E-03	9.47730E-01	2.75840E-02
bSMA	9.76980E-01	1.52200E-02	9.87960E-01	1.07770E-02	9.44860E-01	**2.28050E-02**
bSSA	9.75380E-01	**1.27480E-02**	9.89030E-01	1.03630E-02	9.38600E-01	2.73910E-02
bCSO	9.76670E-01	2.27150E-02	9.90150E-01	9.59290E-03	9.45920E-01	3.59060E-02
BBA	8.87360E-01	8.01780E-02	9.66280E-01	1.92000E-02	8.40620E-01	1.02400E-01
